# Discovery of Novel Isofunctional SARS-CoV‑2 NSP14 RNA Cap Methyltransferase Inhibitors by Structure-Based Virtual Screening

**DOI:** 10.1021/acsmedchemlett.5c00339

**Published:** 2025-08-15

**Authors:** Cindy Meyer, Mayako Michino, David J. Huggins, Aitor Garzia, Jada A. Davis, Michael W. Miller, Nigel Liverton, Hans-Heinrich Hoffmann, J. Fraser Glickman, Julius Nitsche, Oleg Ganichkin, Stefan Steinbacher, Charles M. Rice, Peter T. Meinke, Thomas Tuschl

**Affiliations:** ∇ Laboratory for RNA Molecular Biology, 5929 The Rockefeller University, 1230 York Avenue, New York, New York 10065, United States; ‡ Sanders Tri-Institutional Therapeutics Discovery Institute, 5929 The Rockefeller University, 1230 York Avenue, New York, New York 10065, United States; § Department of Physiology and Biophysics, Weill Cornell Medical College, 1300 York Ave, New York, New York 10065, United States; ∥ Laboratory of Virology and Infectious Disease, 5929 The Rockefeller University, 1230 York Avenue, New York, New York 10065, United States; ⊥ Fisher Drug Discovery Resource Center, 5929 The Rockefeller University, 1230 York Avenue, New York, New York 10065, United States; # PROTEROS Biostructures GmbH, Bunsenstrasse 7a, 82152 Planegg-Martinsried, Germany

**Keywords:** Coronavirus, NSP14, RNA methyltransferase, drug development, structure-based virtual screening, high throughput screening

## Abstract

In early 2020, SARS-CoV-2 spread into a worldwide pandemic, causing more than 7 million deaths. Direct-acting antivirals (DAAs) complementing vaccines and mitigating severe disease in at-risk populations remain important. Here, we used a structure-based virtual screening (SBVS) workflow to identify new SAH-dependent inhibitors of the SARS-CoV-2 RNA cap methyltransferase NSP14. We virtually screened the Enamine and Sigma in-stock screening collections as well as the 3 orders of magnitude larger Enamine REAL make-on-demand compound library, which produced better docking scores and higher virtual hit rates. While biochemical testing of 145 in-stock library compounds yielded a single NSP14-specific inhibitor, 123 chemically synthesized Enamine REAL SBVS compounds contained 10 hits specifically inhibiting NSP14 with half-maximal inhibitory concentrations (IC_50_) below 10 μM. The new compounds were chemically distinct in atomic composition from any NSP14 inhibitors previously identified by conventional biochemical high-throughput screening (HTS) and may serve as starting points to develop novel SARS-CoV-2 DAAs.

## Graphical abstract

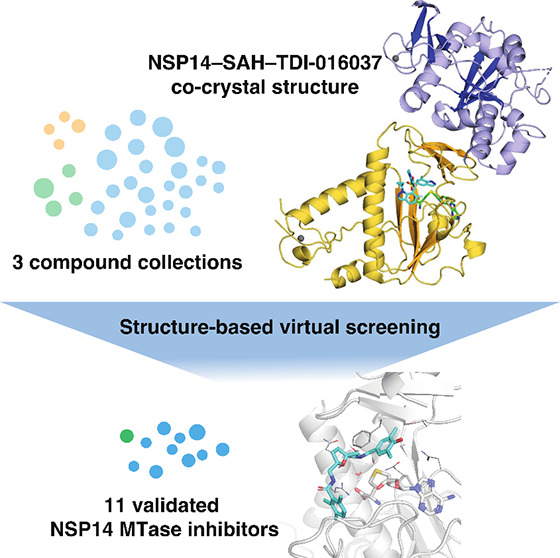

Coronavirus Disease 2019 (COVID-19) is caused by Severe Acute Respiratory Syndrome Coronavirus (SARS-CoV-2),^4^ which triggered an unprecedented global health crisis in early 2020. Although vaccines have been instrumental in controlling the pandemic, their effectiveness has not been uniform–especially among high-risk populations, such as the elderly and immunocompromised.^5^ For the treatment of COVID-19, early global efforts focused on developing small-molecule direct-acting antivirals (DAAs)^1,2^ targeting coronavirus infections. The viral main protease (Mpro) and RNA-dependent RNA polymerase (RdRP) were the first targets for which antiviral drugs were approved,^6^ including nirmatrelvir, remdesivir, and molnupiravir. However, the dynamic evolution of human coronaviruses (hCoVs), driven by vaccination-induced selective pressures^7^ and viral mutations,^8^ underscores the critical need for development of DAA candidates that target novel unexploited pathways in the viral life cycle.

The genetic information on SARS-CoV-2 is contained in a 30-kb single strand of positive-sensed RNA and is replicated in the cytosol. The genome and its subgenomic mRNAs are modified by viral proteins to carry a 5′ cap structure, like eukaryotic mRNAs, required for RNA stability, mRNA translation initiation, and protection from host innate immune responses.^9^ The unmethylated cap structure of the genomic and subgenomic viral RNA is formed cotranscriptionally starting from nascent 5′ triphosphorylated ppp-A-RNA through sequential enzymatic reactions. The viral GpppA-RNA is then methylated at the guanine cap at the N7 position by NSP14 creating the cap-0 structure m^7^GpppA-RNA.^10^ Lastly, NSP16, a nucleoside-2′-O-methyltransferase (MTase), methylates the ribose-2′-OH group of the RNA’s first nucleotide to form the cap-1 structure m^7^GpppA_m_-RNA.^11^ Since the coronaviral RNA cap MTase NSP14 is essential for viral replication^12^ and structurally distinct from commonly known cellular and viral MTases,^13^ it represented an excellent target for DAA development.^14^

We conducted an HTS of 430,376 unique compounds for inhibiting the MTase activity of NSP14. Hit compound RU-0415529 showed an IC_50_ value of 356 nM and was advanced by medicinal chemistry to TDI-015051,^3^ which inhibited viral replication in a transgenic mouse model of SARS-CoV-2 infection with efficacy comparable to the FDA-approved Mpro inhibitor nirmatrelvir.^15^ The animal proof-of-concept (POC) experiments were facilitated by the inhibition of drug-metabolizing CYP enzymes and require future optimization of drug-like properties. Co-crystal structure analyses revealed that TDI-015051 occupied the RNA cap binding pocket of the NSP14 MTase domain, forming a highly selective and highly stable ternary inhibitory complex with the reaction product S-adenosylhomocysteine (SAH).^3^ We decided to exploit our recent crystal structures of SARS-CoV-2 NSP14 in complex with potent inhibitors to identify new compound classes that have the potential for distinct pharmacology and absorption, distribution, metabolism, and excretion (ADME) properties, using the power of virtual screening (VS).

VS of in-stock compound libraries and rapidly growing make-on-demand compound libraries is considered a fast, cost-effective, and environmentally friendly alternative to HTS. Recent advances in large-scale VS methods combined with rapidly increasing compound libraries consisting of more than 100 million compounds have the potential to increase hit rates and potencies.^16,17^ The make-on-demand ultralarge virtual libraries, such as Enamine REAL,^18^ Mcule ultimate,^19^ and WuXi GalaXi,^20^ now access hundreds of million to tens of billion compounds with a predefined path for chemical synthesis. Artificial intelligence (AI)-based virtual screen methods have been developed to screen ultralarge libraries.^21–23^ Incorporation of physics-based free-energy perturbation (FEP) calculation to rescore compounds in the last step of the virtual screening workflow has further enhanced hit rates.^24,25^ For SARS-CoV-2 NSP14, SBVS against the SAM binding site using the Enamine REAL, fragment, and covalent libraries recently identified inhibitors with low μM activities.^26^ However, achieving selectivity over human SAM-dependent MTases remains a challenge for targeting SAM cofactor binding sites.

In the process of optimizing the drug-like properties of TDI-015051,^3^ we derivatized the sulfonamide linkage to a sulfone resulting in various analogs, including TDI-016037 (Figure 1a). TDI-016037 appeared to be a more potent biochemical inhibitor compared to TDI-015051 based on its apparent melting temperature (T_M_) of the NSP14–SAH–drug ternary complex determined by differential scanning fluorimetry (DSF)^27^ (Figure 1b,c, Figure S1a).

**Figure 1:**
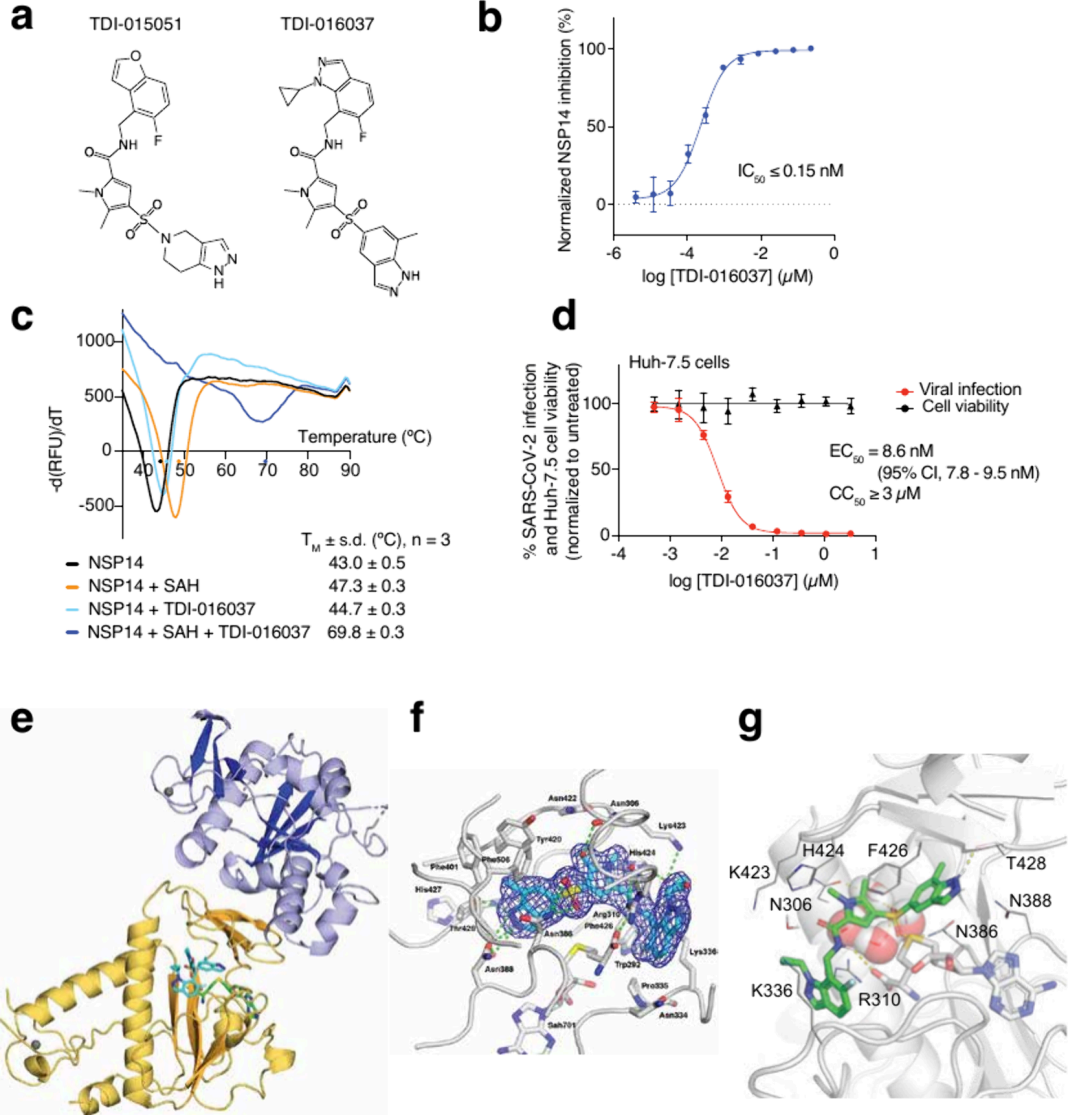
(a) Chemical structures of TDI-015051 and TDI-016037. (b) Inhibition of SARS-CoV-2 NSP14 by TDI-016037; *n* = 3. (c) NSP14 thermal unfolding profiles in the absence or presence of SAH and/or TDI-016037 determined by DSF. Curves are representative of 3 independent measurements. (d) Anti-SARS-CoV-2 activity of TDI-016037 and cytotoxicity in Huh-7.5 cells; *n* = 3; CI, confidence interval. (e) NSP14–SAH–TDI-016037 cocrystal structure (PDB-ID: 9R5T). Subunits of NSP14 are shown as yellow (MTase domain) and blue (exonuclease domain) ribbons. SAH and TDI-016037 are shown as stick models colored according to the chemical atom type (C_TDI‑016037_ in cyan, C_SAH_ in green, N in blue, O in red, S in yellow, and F in pale cyan). (f) NSP14-bound TDI-016037 superimposed with the refined 2Fo-Fc electron density map contoured at the 1.0 σ level. The ligand and neighboring protein side chains are shown as a stick model, colored according to the chemical atom type (C_TDI‑016037_ in cyan, C_NSP14_ in gray, N in blue, O in red, S in yellow, and F in pale cyan). Hydrogen bonds are indicated as green dotted lines. (g) Key protein–ligand interactions formed in the NSP14-SAH-TDI-016037 ternary complex.

The T_M_ of NSP14 alone was 43.0 °C and increased to 47.3 °C upon addition of SAH. Addition of TDI-015051 alone did not increase the T_M_ of NSP14, while addition of TDI-016037 increased the NSP14 T_M_ to 44.7 °C. However, addition of TDI-015051 or TDI-016037 together with SAH to NSP14 dramatically increased the T_M_ values to 64.8 °C or 69.8 °C, respectively, indicating the formation of highly stable NSP14–SAH–drug ternary complexes. TDI-016037 inhibited cell culture SARS-CoV-2 infection with a half-maximal effective concentration (EC_50_) of 8 nM, while no cytotoxicity was detected at ≤ 3 μM compound (Figure 1d).

A NSP14–SAH–TDI-016037 ternary complex cocrystal structure was solved at 2.19 Å resolution (Figure 1e, PDB ID 9R5T) using established methods.^3^ TDI-016037 bound the RNA cap binding site of the MTase domain of NSP14, adjacent to the SAM/SAH binding site (Figure 1f) directly contacting SAH by forming a hydrogen bond between the amide-NH of the compound and the carboxylate of SAH (Figure 1g). This structure was used to computationally discover isofunctional inhibitors of NSP14 by SBVS exploring in-stock and made-on-demand diverse compound libraries (Figure 2).

**Figure 2:**
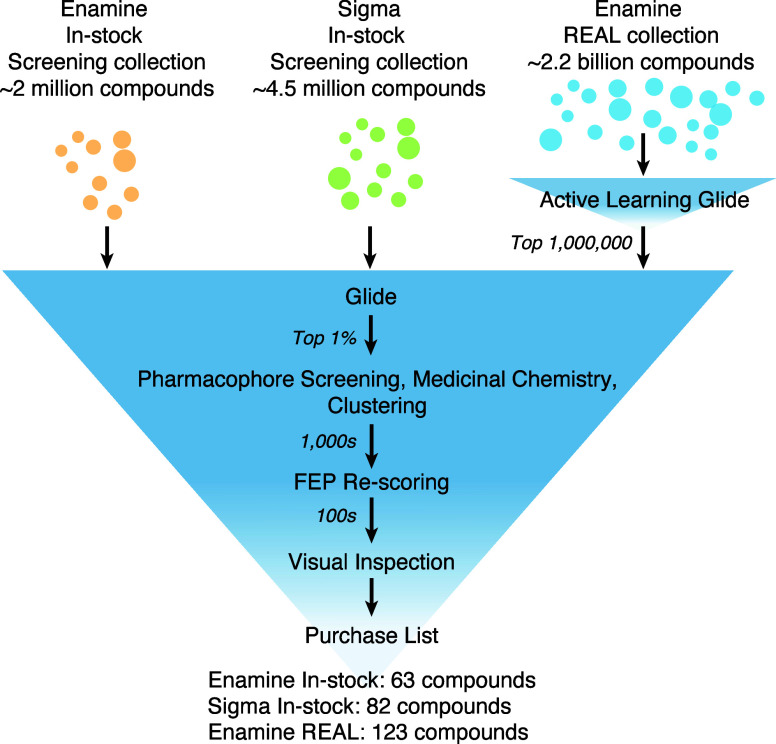
Workflow of the virtual screening process used for the three libraries.

## Retrospective Virtual Screening

To gauge the expected performance of a prospective SBVS, we first evaluated the NSP14–SAH–TDI-016037 cocrystal structure using a retrospective SBVS. The structural model was challenged with discriminating a set of 40 active compounds from 55 inactive compounds (Suppl. Table 1) in the series along with 2,386 property-matched decoys by docking these compounds using Schrödinger’s Glide SP docking program. We found that 36 out of 40 actives scored within the top 5%, which indicated good early enrichment and suggested a promising outcome in a prospective SBVS.

## SBVS of In-Stock Compound Libraries

As a first prospective SBVS, we performed a traditional-scale SBVS with two in-stock compound libraries: (i) a drug-like subset of the Enamine screening collection consisting of approximately 2 million compounds and (ii) a drug-like subset of the Sigma-Aldrich Market Select screening collection consisting of approximately 4.5 million compounds. For each library screen, compounds were docked using the Glide SP docking program in the first step, in which the generated docking grid imposed a hydrogen bond constraint to the carboxylate oxygen atom of SAH. Then, ranked by docking score, the top 1% of compounds (66,000 compounds for the Enamine screen, 124,000 compounds for the Sigma screen), with docking scores between −11.2 to −8.1 for both screens, were selected for postprocessing. Compounds were filtered by several criteria: (i) a pharmacophore model to triage compounds that could form the known key interactions, (ii) a medicinal chemistry filter to remove any compounds with structural alerts, and (iii) clustering to select for chemical diversity. This resulted in a set of approximately 1,000 compounds from each library.

Each set was then rescored by FEP calculations to rerank the compounds using a more rigorous physics-based method to predict the binding free energy. We used a separated topologies approach^28,29^ to calculate the binding free energy of each ligand relative to TDI-016037. Nonbonded interactions between TDI-016037 and each test ligand were set to zero, and the two ligands were restrained to one another for the entirety of all simulations using the six bond, angle, and torsion restraints identified by Boresch.^30^ Transformations to calculate ΔΔG_relative_ were performed in three steps (Figure 3) in both the complex and solvent. The FEP rescoring led to approximately 100 compounds with predicted IC_50_ values lower than 50 μM. The final triaged sets of compounds were visually inspected for medicinal chemistry liabilities and progressibility. From the resulting 63 or 84 compounds, from the Enamine or Sigma libraries, respectively, 63 or 82 compounds were available for purchase and testing (Table 1).

**Figure 3:**
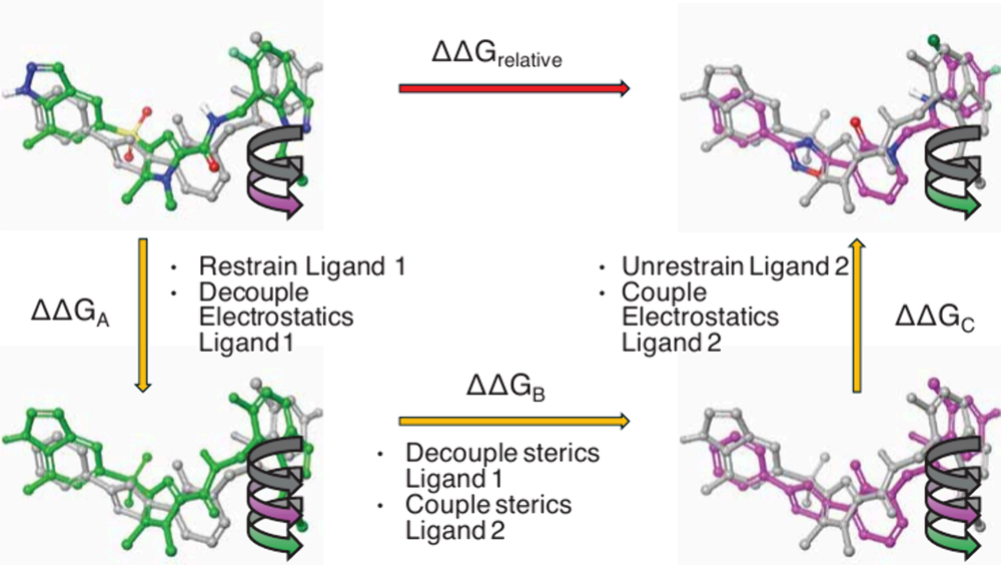
Overview of calculations of ΔΔG_relative_ (solvent only). The black curly arrows represent the six ligand–ligand restraints. The green and magenta curly arrows represent the ligand torsional restraints for ligands 1 (TDI-016037) and 2, respectively. Gray ligands represent decoupled sterics and electrostatics. Monocolor ligands represent decoupled electrostatics. Atom-colored ligands represent coupled sterics and electrostatics.

**Table 1:** SBVS Workflow and Results

**Compound collection**	**Enamine screening**	**Sigma screening**	**Enamine REAL**
Screened compounds	∼2 million	∼4.5 million	∼2.2 billion
Selected from Glide/AL-Glide docking	∼66,000	∼124,000	∼10,000
Selected from pharmacophore and filtering	∼5,000	∼7,000	723
Selected from clustering	∼1,000	∼1,500	NA
Selected from FEP rescoring	84	100	388
Selected by visual inspection	63	84	172
Purchased compounds	63	82	123
Hits (IC_50_ ≤ 10 μM)	1	2	10
Hit rate (%)	1.6	2.4	8.1
NSP14 specific hits (IC_50_ ≤ 10 μM)	0	1	10

One (RU-0833661) and two (RU-0833868 and RU-0833928) compounds from the Enamine or Sigma screening collections showed IC_50_ values of ≤ 10 μM, respectively (Suppl. Tab. 2). We excluded RU-0833661 and RU-0833868, which also inhibited ZIKV NS5 with IC_50_ values of 73.7 or 122 μM, respectively (Suppl. Tab. 2). In summary, RU-0833928 was identified and confirmed as a specific hit by SBVS efforts of the two in-stock compound libraries and an IC_50_ of 9.1 μM for NSP14 (Figure 4). The docked pose of RU-0833928 shows the amide-NH of the compound forming a hydrogen bond with the SAH carboxylate (Figure 5a). Similar to TDI-016037, its sulfone group is predicted to interact with water molecules retained in the binding site. The aromatic benzotriazinone moiety is stacked on top of the SAH-Arg310 salt bridge.

**Figure 4:**
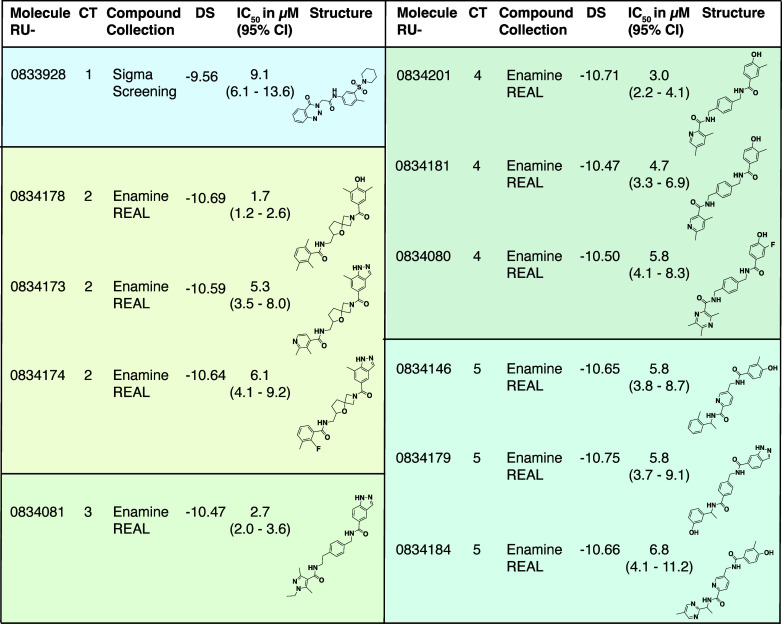
NSP14-specific hits derived from SBVSs of the in-stock Sigma screening and the Enamine REAL compound collections grouped by chemotype (CT). Docking scores (DS), IC_50_ values (*n* = 3) for the inhibition of SARS-CoV-2 NSP14, and chemical compound structures are given. CI, Confidence interval.

**Figure 5:**
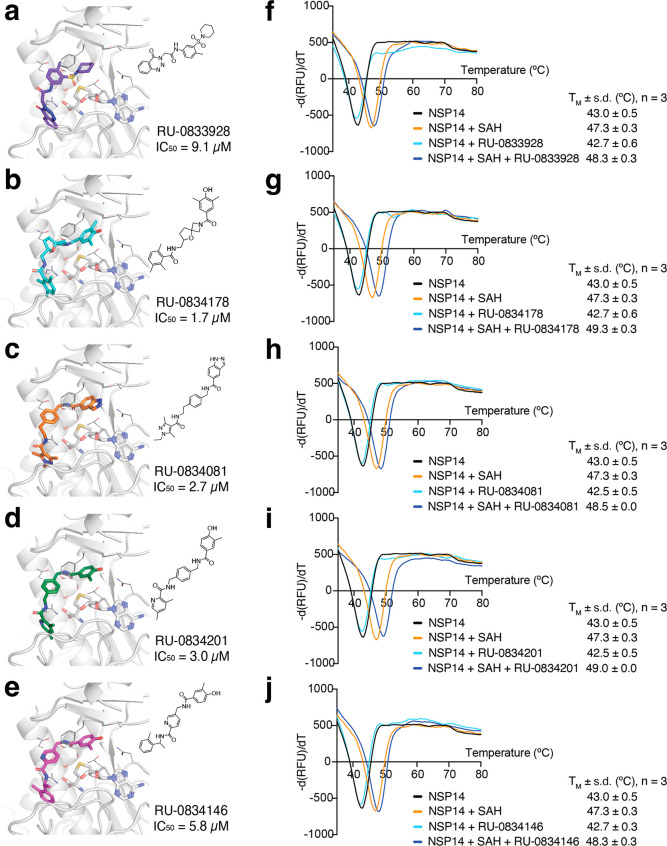
Docked poses of representative hits from the 5 chemotypes identified by SBVS of the in-stock Sigma screening (a) or the Enamine *REAL* compound collection (b-e). Docked poses are shown as colored sticks. NSP14 is depicted as gray ribbons with side chains of key binding site residues in thin gray sticks. SAH is in gray sticks. Created with PyMOL. (f-j) Impact of RU-0833928 (f), RU-0834178 (g), RU-0834081 (h), RU-0834201 (i), or RU-0834146 (j) on the thermal melting profiles of NSP14 in the presence or absence of SAH; *n* = 3.

## SBVS of Ultralarge Compound Library

Encouraged by these results, SBVS was then performed with the approximately 2.2 billion compound-drug-like subset of the Enamine REAL compound library. We used the Schrödinger suite Active Learning Glide (AL-Glide) docking program, which combines ML with docking to efficiently screen ultralarge libraries, in the first step of a screening workflow. The output from this step is one million compounds which are prioritized by the ML model, redocked by Glide SP, and ranked by the docking score. The top 1% of these 1 million compounds (approximately 10,000) had improved docking scores compared to those from the Enamine or Sigma screens, ranging from −12.2 to −10.4. The new compounds were filtered by the pharmacophore model and the medicinal chemistry filter as described above for the Enamine and Sigma screens. This set of 723 compounds was rescored by FEP calculations, which identified 388 compounds with predicted IC_50_ ≤ 50 μM. After visual inspection for medicinal chemistry liabilities and progressability, compounds were clustered by K-means clustering, resulting in a buy-list of 172 compounds, of which 123 were readily synthesized (Table 1). Of these, 10 exhibited *in vitro* anti-NSP14 activity with IC_50_ ≤ 10 μM and also passed all selectivity assays (Table 1, Suppl. Tab. 2). The compounds were grouped into 4 chemotypes based on the core scaffold replacing the sulfone-pyrrole moiety of TDI-016037 (Figure 4).

The docked poses of the most potent analogue of each chemotype shared key interactions with NSP14 binding site residues (Figure 5b-e). Compounds within the same chemotype had consistent binding modes. All four chemotypes had two amides with divergent central moiety, where one amide hydrogen bonds with the SAH carboxylate, and the other amide hydrogen bonds with the water molecules retained in the binding site. The RU-0834178 scaffold has an oxa-azaspiro-octane moiety flanked by two amides (Figure 5b). The RU-0834081 and RU-0834201 scaffolds have 1,4-phenylene-bis-alkylene flanked by two amides, with variable linker length for one of the amide linkages (ethyl linker in RU-0834081, methylene linker in RU-0834201) (Figure 5c, d). The RU-0834146 scaffold has a pyridyl or phenyl moiety flanked by two amides, similar to the RU-0834081 and RU-0834201 scaffolds, but with one of the amides reversed (Figure 5e). Replacing the 7-methyl-indazole of TDI-016037, 4-hydroxybenzamide is a common motif shared by three out of the four scaffolds, and it mimics the hydrogen bonding and aromatic interactions made with Thr428, Asn388, and Phe426. All four chemotypes have an aromatic moiety on the other end of the compound that stacks on top of the SAH-Arg310 salt bridge.

DSF revealed that binding of RU-0833928 (chemotype 1 derived from the Sigma screening collection) and binding of RU-0834173, RU-0834081, RU-0834201, or RU-0834146, the most potent analogs around chemotypes 2, 3, 4, and 5, increased the T_M_ of the NSP14-SAH complex from 47.3 °C to 48.3 °C, 49.3 °C, 48.5 °C, 49.0 °C, or 48.3 °C, respectively, supporting a ternary complex formation as mode of inhibition (Figure 5f-j). None of the inhibitors increased the T_M_ of NSP14 in the absence of SAH.

## Scoring of HTS Hits

To compare the SBVS hits to the experimental HTS hits, we performed docking calculations for 16 original HTS hits identified by robotic screening of our large drug-like chemical library with IC_50_ < 10 μM that also acted by forming an SAH-dependent ternary complex with NSP14 (Suppl. Tab. 3).^3^ The HTS hit RU-0415529, from which the improved TDI-016037 was optimized, had a docking score of −8.04, which is close to the top 1% scoring compounds by SBVS of the in-stock screening libraries. However, the docking scores of the other structurally and chemically distinct 15 HTS hits were all worse than −8.0 and would not have been selected by the SBVS protocol. Despite these hits also forming a ternary NSP14 complex, distinct drug–protein interactions and/or water-mediated interactions may be the reason for this. These observations highlight a key limitation of using a single static protein structure for docking: small conformational differences, such as a single rotamer shift in an amino acid side chain, can lead to steric clashes that prevent the accurate modeling of the binding mode. Consequently, accurately predicting the binding affinity of an experimental HTS hit requires identification of not only its correct binding pose but also the correct protein conformation. This is fundamentally distinct from the problem addressed by docking calculations for SBVS, which aim to identify the correct binding pose and corresponding docking score for a specific protein conformation.

The FEP rescoring calculations performed slightly better in terms of assessing the known HTS hits. We performed FEP calculations on docked models of the 9 uncharged HTS hits. Six of the ligands would have scored in the top 100 for the Sigma and Enamine screens, though only 1 ligand would have scored in the top 100 for the Enamine REAL screen. As for docking, not using the required protein conformation or active site water molecules may also have affected the performance of the FEP calculations. While protein conformational changes can be modeled by FEP calculations, domain shifts, large loop motions, and rotation of some residue side chains are commonly not sampled. In addition, adding or removing water molecules from the binding site can lead to sampling issues.

The importance of the RNA cap MTase activity of CoV NSP14 has been known for more than a decade.^10^ Most SARS-CoV-2 NSP14 inhibitor development efforts targeted the SAM binding site.^31–33^ SAM analogs and nucleoside derivatives often face key limitations, such as poor cell permeability or low selectivity resulting from their structural similarity to enzyme cofactors shared by many enzymes.^31–33^ We recently reported on TDI-015051,^3^ which, unlike inhibitors that displace the universal SAM- or SAH-enzyme-cofactor, occupies the pocket of the MTase-specific substrate of methylation and therefore is highly selective. To discover a more diverse set of drug-like small-molecule NSP14 inhibitors, serving as additional starting points for early stage optimization and diversification of pharmacologic properties, we performed SBVS campaigns of drug-like subsets of two in-stock compound screening collections. We found a significantly higher hit rate with the ultralarge Enamine REAL library, which is consistent with Irwin and Shoichet’s studies reporting that docking scores and hit rates improve with increasing library size.^17,34^ While the workflow for the ultralarge Enamine REAL library used an additional step of Glide Active Learning (Figure 2), the downstream workflow used the same scoring function to select compounds (Glide SP) and subsequent steps including FEP as for the smaller libraries. While achieving an 8.1% hit rate with the Enamine REAL library and discovering novel chemotypes, it should be noted that we used a high-resolution cocrystal structure defining a pharmacophore based on a chemically optimized and highly potent compound originating from a conventional HTS hit and an advanced medicinal chemistry program covering nearly 1,000 derivatives. Hit finding in the absence of a known inhibitor is often more challenging.

Our SBVS approach employed an empirical scoring function designed to approximate the ligand binding affinity based on physical parameters such as van der Waals and electrostatic interactions, hydrogen bonding interactions, and shape complementarity and may not adequately consider conformational strain, desolvation effects, and entropic penalties. Thus, because of the inherent approximations entering docking scores, certain chemotypes may have had artificially good scores that did not necessarily reflect true binding affinity, resulting in false positives. To try and address this, we employed a rigorous postdocking triage process that included rescoring with FEP calculations and manual inspection of binding poses. Using a relative FEP calculation with a separated topology approach, rather than an absolute FEP calculation, allowed us to reduce simulation times and screen more ligands. This step was critical for discriminating promising candidates from artifacts prior to the experimental validation. From 63, 82, or 123 compounds identified from the Enamine screening, Sigma screening, or Enamine REAL ultralarge library, 0, 1, or 10 selective and SAH-dependent inhibitors of NSP14, respectively, underscore the utility of SBVS to identify hit compounds with the potential for distinct pharmacological properties. However, considering the moderate potency of the SBVS hits, medicinal chemistry efforts would be required for optimization, as was the case for the HTS hits. This process could be aided by generative chemistry approaches to expand the chemical space or by exploiting modeling techniques such as QSAR using the available bioactivity/ADME data and docking using the available crystal structures. In terms of the potential for cell permeability and downstream cell-based activity, the SBVS hits were prefiltered for calculated drug-like properties. For the 11 biochemically validated and NSP14-specific SBVS hits, clogP values of compounds were between 1.9 and 3.8 and had 1–4 hydrogen bond donors and 4–7 hydrogen bond acceptors. Molecular weights were between 403.5 and 476.5 g/mol. For comparison, TDI-015051 ^3^ had a clogP value of 1.4, 2 hydrogen bond donors, 5 hydrogen bond acceptors, and a molecular weight of 471.5 g/mol.

Biochemical HTS as well as SBVS require the establishment of experimental assays including expression of recombinant and enzymatically active proteins, which is laborious and perhaps the biggest time factor in early drug discovery. When we conducted a HTS of 430,376 diverse drug-like compounds to identify NSP14 MTase inhibitors, we obtained 16 validated selective hits (Suppl. Tab. 3) at a cost of about 0.5 $ per screening point, including labor. The costs associated with SBVS were an order of magnitude lower than those of our HTS but also yielded fewer biochemically validated hits and less potent starting points (Suppl. Tab. 2) compared to conventional HTS. Disregarding assay development, the time to conduct our HTS took about 8 weeks, followed by a similar wait-time to repurchase and/or resynthesize HTS hit compounds to confirm activity. The wait-time to obtain SBVS compounds was comparable and also took up to 8 weeks. In addition, triaging nonselective HTS hits and eliminating assay interference compounds can be a significant endeavor. However, among the tested SBVS hits, we still identified nonselective compounds and the need for selectivity assays persisted. Overall, we have shown that SBVS approaches can be successfully integrated into experimentally advanced drug discovery programs to provide alternative starting points to optimize pharmacological properties.

## Supplementary Material









## Data Availability

X-ray cocrystal structure factor files have been deposited at RCSB Protein Data Bank (9R5T).

## Notes

Supplementary Figures 1 and 2, Supplementary Tables 4–6, Experimental procedures, details for the preparation of TDI-016037, ^1^H NMR/UPLC of TDI-016037, as well as references of the SI (PDF)
Supplementary Table 1 (XLSX)
Supplementary Table 2 (XLSX)
Supplementary Table 3 (XLSX)

## Abbreviations

DAA: direct-acting antiviral
SBVS: structure-based virtual screening
MTase: methyltransferase
SARS-CoV-2: severe acute respiratory syndrome coronavirus
COVID: coronavirus disease
RdRP: RNA-dependent RNA polymerase
hCoVs: human coronavirus
NSP14: nonstructural protein 14
HTS: high throughput screening
SAM: S-adenosylmethionine
SAH: S-adenosylhomocysteine
FEP: free-energy perturbation
DSF: differential scanning fluorimetry
AL: active learning
DS: docking score
CT: chemotype
RNMT: RNA guanine-7 methyltransferase
RAM: RNMT-activating miniprotein
ML: machine learning
DRC: dose–response curves
ADME: absorption, distribution, metabolism, and excretion
