# Supplementary material for: Discovery of Novel Isofunctional SARS-CoV‑2 NSP14 RNA Cap Methyltransferase Inhibitors by Structure-Based Virtual Screening
Source: ACS Med Chem Lett. 2025 Aug 15;16(9):1789–97. doi: 10.1021/acsmedchemlett.5c00339 (PMC12434519; doi:10.1021/acsmedchemlett.5c00339)
Supplement: Supplementary file 1 [file ml5c00339_si_001.pdf]

Supporting Information

Discovery of novel isofunctional SARS-CoV-2 NSP14 RNA cap methyltransferase inhibitors by structure-based virtual screening

Cindy Meyer[1,#], Mayako Michino[2,#], David J. Huggins[2,3], Aitor Garzia[1], Jada A. Davis[1], Michael W. Miller[2], Nigel Liverton[2], Hans-Heinrich Hoffmann[4], J. Fraser Glickman[5], Julius Nitsche[6], Oleg Ganichkin[6], Stefan Steinbacher[6], Charles M. Rice[4], Peter T. Meinke[2], and Thomas Tuschl[1,*]

[1]Laboratory for RNA Molecular Biology, The Rockefeller University, 1230 York Avenue, New York, NY 10065, USA

[2]Sanders Tri-Institutional Therapeutics Discovery Institute, The Rockefeller University, 1230 York Avenue, New York, NY 10065, USA

[3]Department of Physiology and Biophysics, Weill Cornell Medical College, 1300 York Ave, New York, NY 10065, USA

[4]Laboratory of Virology and Infectious Disease, The Rockefeller University, 1230 York Avenue, New York, NY 10065, USA

[5]Fisher Drug Discovery Resource Center, The Rockefeller University, 1230 York Avenue, New York, NY 10065, USA

[6]PROTEROS Biostructures GmbH, Bunsenstrasse 7a, 82152 Planegg-Martinsried, Germany

*Correspondence to: ttuschl@rockefeller.edu

[#]Authors contributed equally to this work.

Table of Contents

Pages 3 – 4	Supplementary Figures 1 and 2
Page 5	Supplementary Table 4: Cost comparison of HTS and SBVS
Page 6	Supplementary Table 5: Data collection and processing statistics for the crystallographic structure of SARS-CoV-2 NSP14 in complex with SAH and TDI-016037
Page 7	Supplementary Table 6: Refinement statistics for crystallographic structure of SARS-CoV-2 NSP14 in complex with SAH and TDI-016037.
Pages 8 – 16	Experimental procedures
Pages 17 – 24	Preparation of TDI-016037
Page 25	^{1}H NMR/UPLC of TDI-016037
Page 26	References Supporting Information

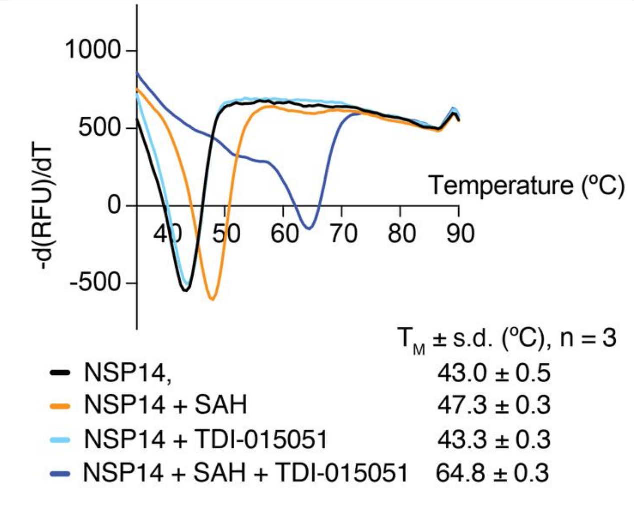

Supplementary Figure 1: NSP14 (1 µM) thermal unfolding profiles over a temperature range of 35 °C to 95 °C in absence or presence of SAH (25 µM) and/or TDI-015051 (20 µM) determined by differential scanning fluorimetry (DSF) illustrated in the first derivative mode. The melting temperature (T_M) corresponds to the inflection point and is visualized as the peak of the curve. Profiles are representative of 3 independent experiments.

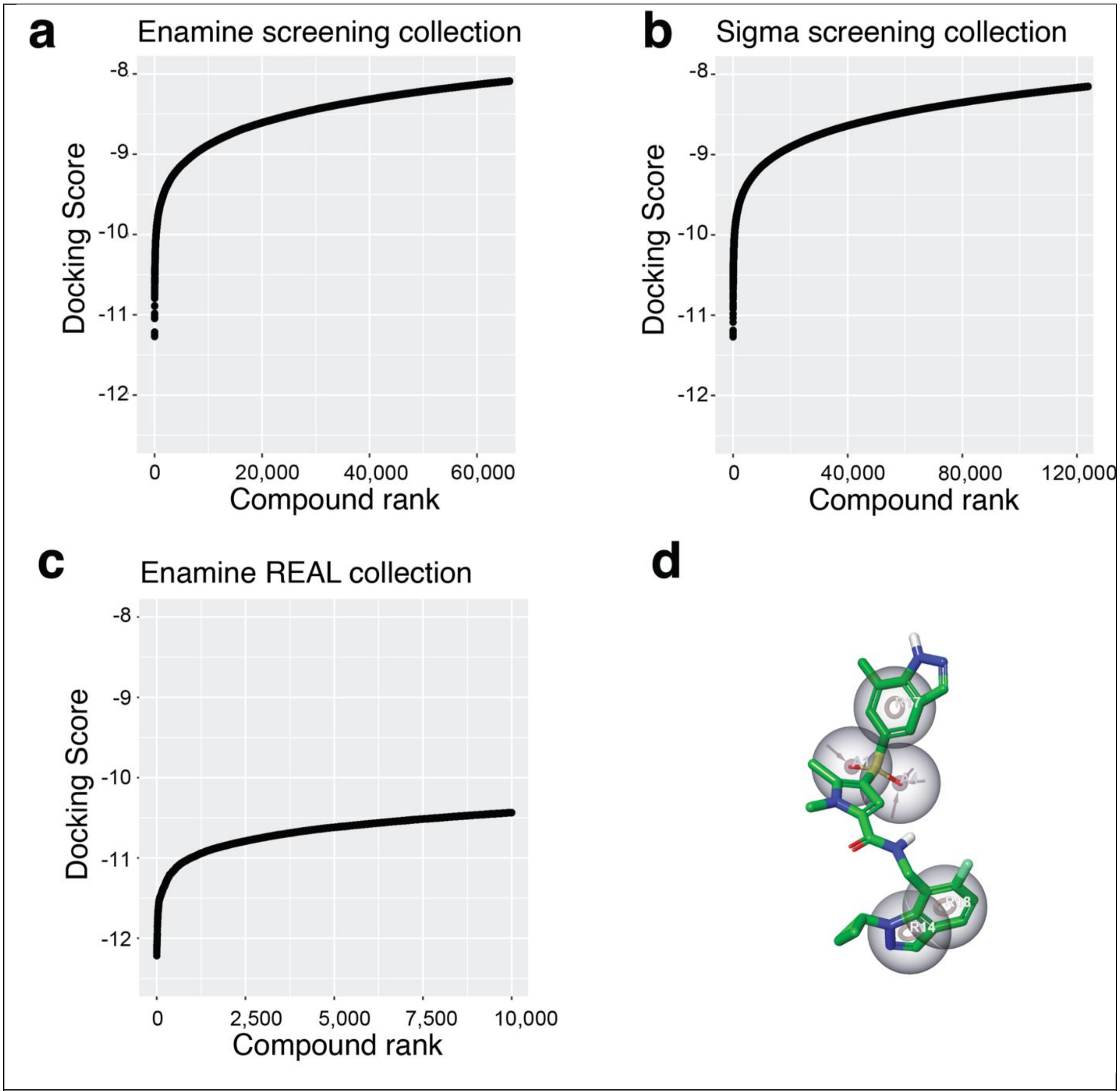

Supplementary Figure 2: Docking scores plotted against compound rank for the Enamine screening collection **(a)**, Sigma screening collection **(b)**, and Enamine REAL make-on-demand ultra-large screening library **(c)**. **d)** Pharmacophore model used in the post-processing step of the VS workflow. The five features defining the pharmacophore model are shown in gray spheres. TDI-016037 is represented in green sticks.

Supplementary Table 4. Cost comparison of HTS and SBVS

**Screening method**	**HTS**	**SBVS**		
**Library**		**Enamine screening**	**Sigma screening**	**Enamine REAL Ultra large**
Resource estimate	$260,000	$13,000	$16,000	$41,000
Screened compounds	430,376	~2,000,000	~4,500,000	~2,200,000,000
Compounds followed up	378	63	82	123
Validated hits	18	1	2	10
Validated hit rate (%)	4.8	1.6	2.4	8.1

Supplementary Table 5: Data collection and processing statistics for the crystallographic structure of SARS-CoV-2 NSP14 in complex with SAH and TDI-016037.

**Ligand**	**TDI-016037-NX-1**
X-ray source	PXII/X10SA (SLS[1])
Wavelength [Å]	1.0000
Detector	Dectris EIGER2 Si 16M
Temperature [K]	100
Space group	P 2_1
Cell: a; b; c; [Å]	67.24; 100.95; 90.25
α; β; γ; [°]	90.0; 108.1; 90.0
Resolution [Å]	1.89 (2.14-1.89)
Unique reflections	58473 (2924)
Multiplicity	4.5 (4.8)
Spherical completeness [%]	64.2 (10.4)
Ellipsoidal completeness [%]	94.3 (67.3)
R_{pim} [%][6]	3.8 (61.1)
R_{sym} [%][3]	7.1 (121.0)
R_{meas} [%][4]	8.1 (136.0)
$CC_{1/2}$ [%]	99.50 (58.00)
Mean(I)/sd[5]	10.0 (1.6)

[1] SWISS LIGHT SOURCE (SLS, Villigen, Switzerland)

[2] values in parenthesis refer to the highest resolution bin.

[3] $$Rsym = \frac{\sum_h \sum_i^{n_h} \left| \hat{I}_h - I_{h,i} \right|}{\sum_h \sum_i^{n_h} I_{h,i}} \quad \text{with} \quad \hat{I}_h = \frac{1}{n_h} \sum_i^{n_h} I_{h,i}$$

where $I_{h,i}$ is the intensity value of the ith measurement of h

[4] $$Rmeas = \frac{\sum_h \sqrt{\frac{n_h}{n_h - 1}} \sum_i^{n_h} \left| \hat{I}_h - I_{h,i} \right|}{\sum_h \sum_i^{n_h} I_{h,i}} \quad \text{with} \quad \hat{I}_h = \frac{1}{n_h} \sum_i^{n_h} I_{h,i}$$

where $I_{h,i}$ is the intensity value of the ith measurement of h

[5] calculated from independent reflections

[6] Precision-indicating $$Rpim = \frac{\sum_h \sqrt{1/(N-1)} \left| I_{hl} - \langle I_h \rangle \right|}{\sum_h \langle I_h \rangle}$$

Supplementary Table 6: Refinement statistics for crystallographic structure of SARS-CoV-2 NSP14 in complex with SAH and TDI-016037.

**Ligand**	**TDI-016037-NX-1**
Resolution [Å]	54.05-1.89
Number of reflections (working /test)	57029 / 1444
R_{cryst} [%]	18.9
R_{free}[%][2]	22.7
Total number of atoms:	
Protein	6918
Water	343
Ligand	74
Imidazole	25
S-adenosyl-l-homocysteine	52
Sulfate	10
Chloride	4
Zinc	7
Isopropyl alcohol	16
1,2-Ethanediol	52
Average B-factors	
Protein	57.6
Ligand	38.7
Water	51.9
Other atoms	63.7
Deviation from ideal geometry: [3]	
Bond lengths [Å]	0.006
Bond angles [°]	1.44
Bonded B's [$Å^2$] [4]	3.2
Ramachandran plot: [5]	
Favoured [%]	97.49
Allowed [%]	2.39
Outliers [%]	0.12
Molprobity score[5]	0.92
Molprobity clashscore[5]	1.00

[1] Values as defined in REFMAC5, without sigma cut-off
[2] Test-set contains 2.5% of measured reflections
[3] Root mean square deviations from geometric target values
[4] Calculated with MOLEMAN
[5] Calculated with Molprobity

Experimental Procedures

Requests for resources and reagents should be directed to Thomas Tuschl (ttuschl@rockefeller.edu). Plasmids were deposited with Addgene.

Cell lines and viral strain

Huh-7.5 were generated from Huh-7 hepatoma cells (*Homo sapiens*; sex: male, liver epithelial)[1] and cultured in Dulbecco's Modified Eagle Medium (DMEM) supplemented with 1% nonessential amino acids (NEAA) and 10% fetal bovine serum (FBS) at 37 °C and 5% CO_2. Cell lines were tested negative for contamination with mycoplasma. SARS-CoV-2 (strain: USA-WA1/2020) was obtained from BEI Resources (NR-52281).

Construction of plasmids for recombinant protein expression

The generation of the expression plasmids pRSF-Duet-His_6-SUMO-SARS-CoV-2-NSP14, pRSF-Duet-His_6-SUMO-hCoV-229E-NSP14, pRSF-Duet-His_6-SUMO-hCoV-NL63-NSP14, pRSF-Duet-His_6-SUMO-ZIKV-NS5-MTase, and pRSF-Duet-His_6-SUMO-hRNMT-RAM has previously been described[2].

Expression and purification of recombinant proteins from E. coli

E. coli Rosetta™2(DE3)pLysS cells (Novagen) harboring either of the pRSF-Duet-His_6-sumo expression constructs were grown in multiples of 1 l Luria-Bertani medium supplemented with 50 mg/ml kanamycin at 37 °C to an OD_{600} of 0.6. After induction with 0.5 mM isopropyl β-D-1-thiogalactopyranoside, cells were grown overnight at 18 °C. Cell pellets were collected via centrifugation at 3,500*g* for 20 min and stored at -80 ºC until usage.

Cell pellets were thawed on ice for 30 min (if previously frozen) and resuspended in 3-5 volumes lysis buffer (50 mM Tris-HCl, pH 7.5, 500 mM NaCl, 20 mM imidazole, 10% glycerol, 0.2% Triton X-100, 1 mg/ml lysozyme). After incubation for 30 min on ice, the lysate was homogenized by ultrasonication (48 W, 10 s sonication, 40 s pause, 2 min total sonication time). After centrifugation at 35,000*g* for 60 min, the supernatant was filtered through a 0.45 µm filter and mixed with cOmplete His-Tag purification resin (Roche) for incubation at 4 ºC overnight. The mixture was loaded onto a gravity flow column. The target protein was eluted using lysis buffer supplemented with 500 mM imidazole. The eluted protein was incubated with ULP1 (0.5 µg/ml

final concentration, lab stock) during dialysis at 4 °C overnight against a buffer containing 50 mM Tris-HCl, pH 7.5, 20 mM imidazole, 500 mM NaCl, and 1 mM dithiothreitol (DTT). To remove the His_6-SUMO tag, the protein sample was mixed with cOmplete His-Tag purification resin (Roche), incubated for 2 h at 4 ºC, and loaded onto a gravity flow column. Since the His_6-SUMO tag bound to the column material, the flow-through was collected containing untagged target protein. The target protein was further purified by a Superdex200 10/300 gel filtration column (GE Healthcare) in buffer (20 mM Tris-HCl, pH 7.5, 500 mM NaCl, 1 mM DTT). Fractions were analyzed by SDS-PAGE and Coomassie staining. Protein-containing fractions were pooled, flash-frozen in liquid nitrogen, and stored at -80 °C.

Biochemical SARS-CoV-2 NSP14 activity assay at 37 ºC

Recombinant SARS-CoV-2 NSP14 (0.3 nM) was incubated with GpppA (3 µM) and SAM (5 µM) in 1X NSP14 reaction buffer (50 mM Tris-HCl, pH 7.5, 6 mM KCl, 1.25 mM $MgCl_2$, 1 mM DTT, and 0.01% Tween-20) in a total reaction volume of 16 µl in absence or presence of increasing inhibitor compound concentrations for 45 min at 37 ºC. SAH production was monitored using the bioluminescence-based MTase-Glo™ Methyltransferase Assay kit (Promega) following manufacturer's instructions. The luminescence signal of each reaction was recorded in relative light units (RLUs) using a Synergy Neo plate reader (BioTek, Winooski, VT). IC_{50} values were calculated using the GraphPad Prism software (Version 10.1.1) and a four-parameter logistic regression. IC_{50} values (mean from at least 3 independent experiments) were calculated using the GraphPad Prism software (Version 10.1.1) and a four-parameter logistic regression.

Biochemical activity assays for the human RNMT-RAM complex and ZIKV NS5 MTases

Activity assays were performed in 1x NSP14 reaction buffer. For hRNMT-RAM, we used 15 nM enzyme, 2.5 µM GpppG and 2 µM SAM. For ZIKV NS5, we used 25 nM enzyme, 3 µM GpppAGUUGUUA[3], and 5 µM SAM. The enzymatic reaction was initiated with the addition of the enzyme in assay buffer and incubated at RT or 37 ºC for 45 min, respectively. SAH was detected using the MTase-Glo™ assay.

Differential scanning fluorimetry (DSF)

Unless otherwise indicated, NSP14 (1 μM) was incubated alone or with single or combinations of ligands (20 μM each) in 1x NSP14 reaction buffer (50 mM Tris-HCl, pH 7.5, 6 mM KCl, 1.25 mM $MgCl_2$, 1 mM DTT, and 0.01% Tween-20) in a final volume of 50 µl with a final DMSO concentration of 1%. A 1% DMSO vehicle control was included in each measurement. SYPRO orange (ThermoFisher, S6650, 5,000X) was added to each reaction at a final concentration of 5X. 10 µl of samples were transferred to wells of a 384-well qPCR plate before standard thermocycling (25 – 95 °C at +1 °C/min). The recorded fluorescence profile was displayed as brightness and plotted against the temperature. The resulting unfolding profiles were automatically analyzed for inflection points, which corresponded to the melting temperature (T_M).

Synthesis of TDI-016037

See supporting information for the synthesis and characterization of TDI-016037.

Crystallization and NSP14-SAH-TDI-016037 co-crystal structure determination

Crystallization procedure, data collection and structure determination: Protein production and crystallization of SARS-CoV-2 NSP14 in complex with SAH and TDI-014988, a lower affinity RU-0415529 analog, was performed as described previously[2] . For exchange of ligand TDI-014988 to TDI-016037, crystals were soaked at saturation concentration with TDI-016037 for three days at 4 °C. Crystals were flash cooled in liquid nitrogen after being passed through a cryoprotectant consisting of well buffer supplemented with 30% ethylene glycol and 1 mM TDI-016037. Data collection and structure determination were performed as described previously[2]. Processing statistics are summarized in Suppl. Tab. 5. The Ramachandran plot of the final model calculated with Molprobity[4] shows 97.49% of all residues in the favored region and 2.39% in the allowed region. The residue Glu453(A) is an outlier in the Ramachandran plot. It is either defined by the electron density or could not be modelled in another sensible conformation. Statistics of the final structure and the refinement process are listed in Suppl. Tab. 6.

SARS-CoV-2 viral infection assay

The SARS-CoV-2 viral infection assay was performed as previously described[2] .

Preparation of compound libraries

The in-stock Enamine and Sigma Aldrich Market Select screening collections (version 2024q1) were obtained from the Phase database distributed by Schrödinger. Properties for these databases are available online:

https://www.schrodinger.com/wp-content/uploads/2024/04/2024q1_sigma_report.pdf
https://www.schrodinger.com/wp-content/uploads/2024/04/2024q1_enamine_report.pdf

The Phase database consists of compound structures in SDF format run through Schrödinger suite LigPrep to generate stereoisomers, tautomers, and charged states using Epik. Database subsets are provided for drug-like, lead-like, near-drug, and fragment compounds, classified based on properties calculated on a single, neutralized and desalted representation. The drug-like subset has the properties: 250 g/mol ≤ MW ≤ 500 g/mol, -1 ≤ AlogP ≤ 4, 50 Å ≤ PSA ≤ 130 Å, ≤ 10 hydrogen bond acceptors, ≤ 5 hydrogen bond donors, ≤ 3 chiral centers, ≤ 10 rotatable bonds. The Enamine REAL compound library was downloaded in SMILES format from the Enamine website https://enamine.net/compound-collections/real-compounds/real-database. The version downloaded in June-August 2024 consisted of 6.5 billion compounds. The drug-like subset of the library was prepared by Schrödinger, based on the same classification criteria as with the in-stock screening collection subsets, and consisted of ~2.2 billion compounds.

Protein preparation and molecular docking of in-stock compound screening collections with Glide
The NSP14-SAH-TDI-016037 co-crystal structure was prepared using the Protein Preparation Workflow in Maestro with default settings (release 2024-2, Schrödinger LLC, New York, NY). A docking grid was generated from the prepared structure, centered on the TDI-016037 compound, and with all default parameters, except keeping the four water molecules resolved in the co-crystal structure that mediate the interactions between the protein and the sulfone of the compound. Compounds were docked using Glide SP (standard precision mode) with the generated docking grid imposing a hydrogen bond constraint to the carboxylate oxygen atom of SAH, and default parameters (release 2023-2, Schrödinger, LLC, New York, NY).

Preparation of compound set for retrospective virtual screening

Representative sets of 40 'actives' and 55 'inactives' were selected from 1,097 compounds in the TDI-015051 series. Compounds with a biochemical IC_{50} less than 1 μM in the MTase assay were classified as 'actives', while compounds with a biochemical IC_{50} greater than 1 μM were classified as 'inactives'. 2,386 property-matched decoys for these 40 actives were generated using DUDe[5]. Compounds are listed in Suppl. Table 1.

AL-Glide

AL-Glide (release 2024-2, Schrödinger, LLC, New York, NY), an ML-enhanced docking protocol in Schrödinger suite that allows for efficient large-scale screening of ultra-large libraries such as the Enamine REAL collection [6], was used to screen the drug-like subset of the Enamine REAL library with default parameters. Briefly, the AL-Glide program performed three iterations of training, with 50,000 compounds in each iteration, to build ML models to predict docking scores. The top 1 million compounds were then re-scored at the end with Glide SP. Compounds were prepared on the fly within the program using LigPrep to account for all relevant tautomeric and ionization states at pH 7.0 ± 1.0, and to enumerate the stereoisomers for structures bearing stereocenters with non-explicit chirality, up to a maximum of 16. The screen was run on 500 CPUs.

Post-processing

After the docking step in the SBVS workflow, the compounds were post-processed by several filters. The docked poses of the compounds were first filtered for matching to a pharmacophore model using the Schrödinger suite Phase ligand screening program. The pharmacophore model represented key interactions formed by TDI-016037 and consisted of five features: three aromatic sites on either side of the sulfone group and two hydrogen bond acceptor sites on the oxygen atoms of the sulfone group (Suppl. Fig. 2d). At least four features needed to match for the molecule to pass. This first filter resulted in ~10,000 and ~16,500 compounds when screening the in-stock Enamine and Sigma screening collections, respectively, and ~1,000 compounds when screening the Enamine REAL collection. Triaged compounds were then filtered for medicinal chemistry structural alerts. The structural alerts were obtained by leveraging Postera's Manifold (https://app.postera.ai) MedChem alerts and consisted of 1,936 SMARTS patterns with the minimum and maximum number of allowed counts. This second filter resulted in ~5,000 and ~7,000 compounds for the in-stock Enamine and Sigma screens, respectively, and 723 compounds

for the Enamine REAL screen. Finally, the triaged in-stock Enamine and Sigma screening collections compounds were clustered to obtain a diverse set of 20% (~1,000-1,500 compounds) using the Schrödinger suite Hit Analyzer panel.

FEP re-scoring

The predicted binding modes from Glide docking were used as the basis for free-energy calculations to predict the binding affinities. Rather than an absolute binding free energy calculation, where the two end states are an apo protein and a ligand-bound complex[7–11], we exploited the existence of an inhibitor with a known binding mode and affinity to perform a relative binding free energy calculation by using a separated topologies approach[12,13]. The protein was parametrized using the AMBER ff15ipq forcefield[14], water molecules were parametrized using the TIP3P water model[15] , and ligands (including SAH) were parametrized using the GAFF 2.11 forcefield[16] with AM1-BCC charges[17]. In previous studies, this combination was found to yield good results in the context of both absolute and relative binding free energy calculations[8,18] . To provide a buffer between the solute and the edge of the rhombic dodecahedral periodic boxes, solvent and complex systems were padded with 20.0 Å and 5.0 Å of solvent respectively. Systems were neutralized and the ionic strength was set to 150 mM with Na^+ and Cl^- ions. Non-bonded interactions between the two ligands were set to zero and the two ligands were restrained to one another for the entirety of all simulations using the six restraints identified by Boresch[19] . We were careful to choose restraining atoms that are part of a relatively rigid framework such that the thermodynamic cycle is close to exact. In this case, the dynamics of the two ligands do not affect one another, allowing for calculation of the relative binding affinity. The restraints were selected and calculated using an automated protocol as follows:

1. Find the heavy atom in ligand 1 that is closest to the centroid of the molecule. This is atom LA1
2. Find the heavy atom in ligand 2 that is second closest to LA1. This is atom LB1
3. Identify all ligand 1 atoms up to two bonds away from LA1 (name this set M)
4. Iterate over all M atoms and calculate the angle m-LA1-LB1
5. Select the m which yields the angle closest to 90 degrees. This is atom LA2
6. Iterate over all remaining M atoms and calculate the angle m-LA2-LA1

7. Select the m which yields the angle closest to 90 degrees. This is atom LA3
8. Identify all ligand 2 atoms up to two bonds away from LB1 (name this set N)
9. Iterate over all N atoms and calculate the angle n-LB1-LA1
10. Select the n which yields the angle closest to 90 degrees. This is atom LB2
11. Iterate over all remaining N atoms and calculate the angle n-LB2-LB1
12. Select the n which yields the angle closest to 90 degrees. This is atom LB3

The force constants used for the ligand-ligand restraints were 20.0 kcal/mol/$Å^2$ for the bonds, 250.0 kcal/mol/rad^2 for the angles, and 250.0 kcal/mol for the dihedrals. LA1-LB1 bond distances ranged from 0.69 Å to 2.99 Å. Ligand heavy-atom/heavy-atom torsion angles were also restrained for some portions of the process to aide convergence. The force constants used for the ligand torsional restraints was 100.0 kcal/mol. Equivalent ligand torsional restraints were applied in the solvent and complex such that the ends states are the same and the binding free energies are not affected. Six calculations were performed to estimate each relative binding free energy. This comprised three calculations in each of the solvent and complex: (A) restraining ligand 1 (TDI-016037) torsional restraints and decoupling ligand 1 electrostatics, (B) decoupling ligand 1 sterics and coupling ligand 2 sterics, and (C) unrestraining of ligand 2 torsional restraints and decoupling ligand 2 electrostatics. These steps are shown in Figure 3. The first step ($\Delta\Delta G_A$) does not depend on the identity of ligand 2 and so was calculated as the average of 1,000 calculations (with different ligand 2 structures to confirm lack of dependence). The standard deviations for $\Delta\Delta G_A$ were 0.5 kcal/mol and 0.7 kcal/mol for the solvent and complex legs respectively, illustrating the lack of dependence.

The free energy calculations were performed using OpenMM version 7.2[20] with the OpenMMTools toolkit for Hamiltonian replica exchange. NPT simulations were conducted at 300 K and 1 atm using a Monte Carlo barostat. Simulations were performed with a timestep of 4.0 fs using hydrogen mass repartitioning and a hydrogen mass of 4 AMU[21]. Electrostatics were modelled with PME[22] and van der Waals were modelled using a nonbonded cutoff of 10.0 Å. Bonds to hydrogen were constrained and water molecules were modelled as rigid. To avoid the numerical instabilities referred to as end point catastrophes that occur when ligands approach the fully decoupled state, OpenMMTools employs a softcore function[23]. Default parameters were used for softcore_alpha (0.5), softcore_a (1), softcore_b (1), softcore_c (6), softcore_beta (0.0),

softcore_d (1), softcore_e (1), and softcore_f (2). We should note that this protocol does not currently allow for the testing of ligands with a change in formal charge and so such ligands were excluded from further consideration. Free energy changes were calculated using MBAR[24] with 12 lambda windows. The exact lambda values for the electrostatics, sterics, and restraints within each lambda window is described by the lambda schedule. We used linear lambda schedules for the sterics and electrostatics. We found that sigmoidal lambda schedules for the restraints with an exponent of 3.5 enhanced the mixing of the alchemical states for the HREX and improved convergence of the free energy. For each lambda window, initial simulations were performed with 500 MD steps per iteration for 50 equilibration iterations (0.1 ns) and 250 production iterations (0.5 ns). Simulations were extended by 2000 steps (4.0 ns) for the top scoring 250 (Sigma and Enamine) or 400 (Enamine REAL) molecules to yield the final predictions. Predicted binding free energies were calculated by combining the final predictions with the experimental activity of TDI-016037. The top-scoring 100 compounds from each library were then selected for purchase.

Preparation of TDI-016037

Preparation of 1-cyclopropyl-6-fluoro-1*H*-indazol-7-yl)methanamine

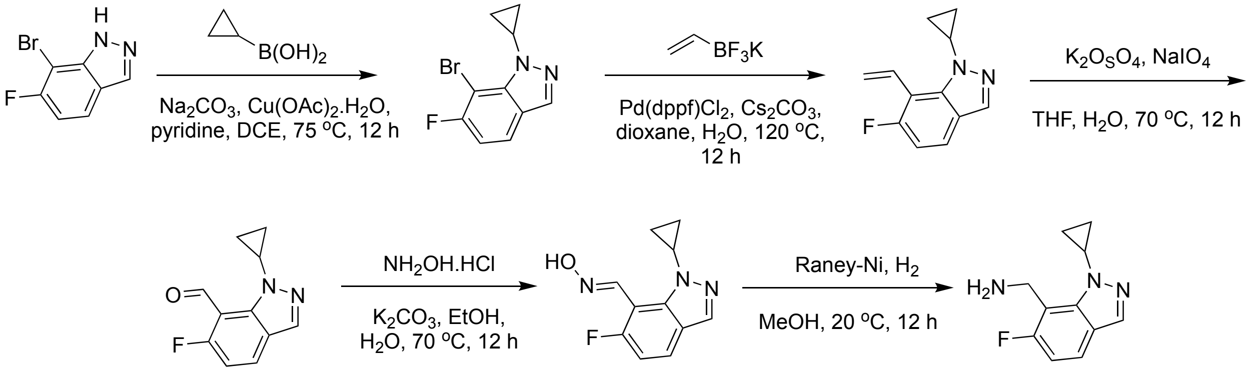

Step 1

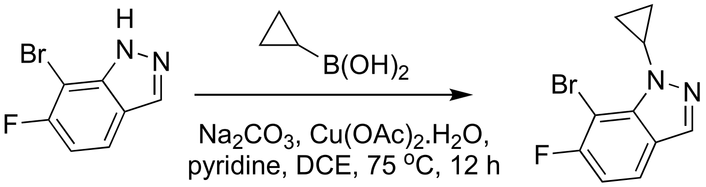

To a solution of 7-bromo-6-fluoro-1H-indazole (300 mg, 1.40 mmol, 1 eq) in DCE (6 mL) was added Na_2CO_3 (296 mg, 2.79 mmol, 2 eq), $Cu(OAc)_2.H_2O$ (279 mg, 1.40 mmol, 1 eq), cyclopropylboronic acid (144 mg, 1.67 mmol, 1.2 eq) and pyridine (110 mg, 1.40 mmol, 1 eq). The mixture was stirred at 75 °C for 12 h under a O_2 atmosphere. The mixture was poured into H_2O (10 mL), and the mixture was extracted with DCM (10 mL x 3). The combined organic layer was washed with brine (10 mL), dried over Na_2SO_4, and filtered. The filtrate was concentrated under reduced pressure. The residue was purified by column chromatography (SiO_2, petroleum ether/ethyl acetate gradient = 1/0 to 20/1) to furnish 7-bromo-1-cyclopropyl-6-fluoro-1H-indazole (190 mg, 0.745 mmol, 53 % yield) as a white solid. LCMS (MH^+) 255.1.

Step 2

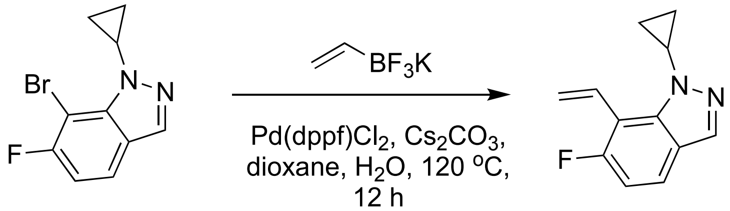

A mixture of 7-bromo-1-cyclopropyl-6-fluoro-1H-indazole (90 mg, 0.35 mmol, 1 eq), trifluoro(vinyl)-λ^4-borane, potassium salt (57 mg, 0.42 mmol, 1.2 eq), $Pd(dppf)Cl_2$ (26 mg, 0.035 mmol, 0.1 eq), Cs_2CO_3 (230 mg, 0.706 mmol, 2 eq) in dioxane (2 mL) and H_2O (0.2 mL) was degassed and purged with N_2 for 3 times. The mixture was stirred at 120 °C for 12 h under a N_2 atmosphere. The reaction was diluted with H_2O (10ml), and the mixture was extracted with EtOAc (15 mL x 3). The combined organic layers were washed with brine (20 mL), dried over Na_2SO_4, and filtered. The filtrate was concentrated under reduced pressure to give a residue. The residue was purified by column chromatography (SiO_2, petroleum ether/ethyl acetate

gradient = 1/0 to 4/1) to furnish 1-cyclopropyl-6-fluoro-7-vinyl-1H-indazole (0.100 g, crude) as a white solid. LCMS (MH^+) 203.2.

Step 3

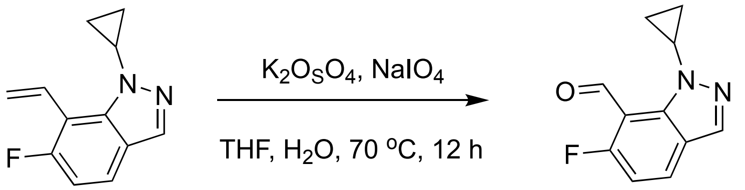

A mixture of 1-cyclopropyl-6-fluoro-7-vinyl-1H-indazole (30 mg, 0.14 mmol, 1 eq), sodium periodate (159 mg, 0.742 mmol, 5 eq), potassium osmate (4.4 mg, 0.12 mmol, 0.08 eq) in THF (1 mL) and H_2O (0.2 mL) was degassed and purged with N_2 for 3 times. The mixture was stirred at 70 °C for 12 h under a N_2 atmosphere. The mixture was diluted with H_2O (10ml), and the mixture was extracted with EtOAc (15 mL x 3). The combined organic layers were washed with brine (20 mL), dried over Na_2SO_4, and filtered. The filtrate was concentrated under reduced pressure to give a residue. The residue was purified by column chromatography (SiO_2, petroleum ether/ethyl acetate gradient = 1/0 to 3/1) to furnish 1-cyclopropyl-6-fluoro-1H-indazole-7-carbaldehyde (40mg) as a white solid. LCMS (MH^+) 205.2.

Step 4

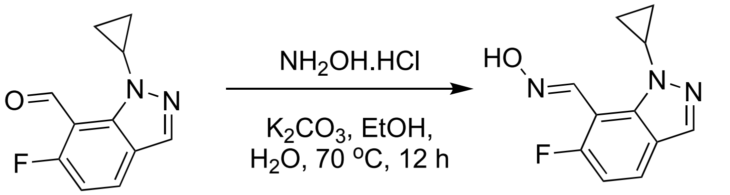

A mixture of 1-cyclopropyl-6-fluoro-1H-indazole-7-carbaldehyde (40 mg, 0.20 mmol, 1 eq), hydroxylamine hydrochloride (44 mg, 0.63 mmol, 3.2 eq), K_2CO_3 (41 mg, 0.29 mmol, 1.5 eq) in EtOH (1 mL) and H_2O (1 mL) was degassed and purged with N_2 for 3 times. The mixture was stirred at 70 °C for 12 h under a N_2 atmosphere. The mixture was diluted with H_2O (10ml), and the mixture was extracted with EtOAc (15 mL x 3). The combined organic layers were washed with brine (20 mL), dried over Na_2SO_4, and filtered. The filtrate was concentrated under reduced pressure furnish (E)-1-cyclopropyl-6-fluoro-1H-indazole-7-carbaldehyde oxime (40 mg, crude) as a white solid. LCMS (MH^+) 220.2.

Step 5

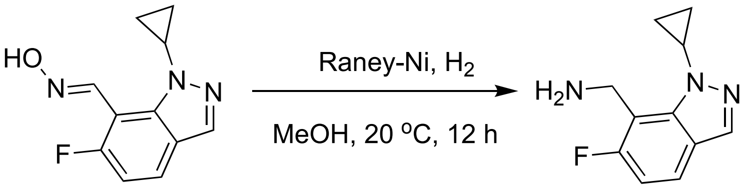

To a solution of (E)-1-cyclopropyl-6-fluoro-1H-indazole-7-carbaldehyde oxime (40 mg, 0.18 mmol, 1 eq) in MeOH (1 mL) was added Raney-Ni (40 mg) under a N_2 atmosphere. The

suspension was degassed and purged with H_2 for 3 times. The mixture was stirred under H_2 (15 Psi) at 20 °C for 12 h. The mixture was filtered and concentrated under reduced pressure to give (1-cyclopropyl-6-fluoro-1*H*-indazol-7-yl)methanamine (15 mg, crude) as a white solid. LCMS (MH^+) 206.3.

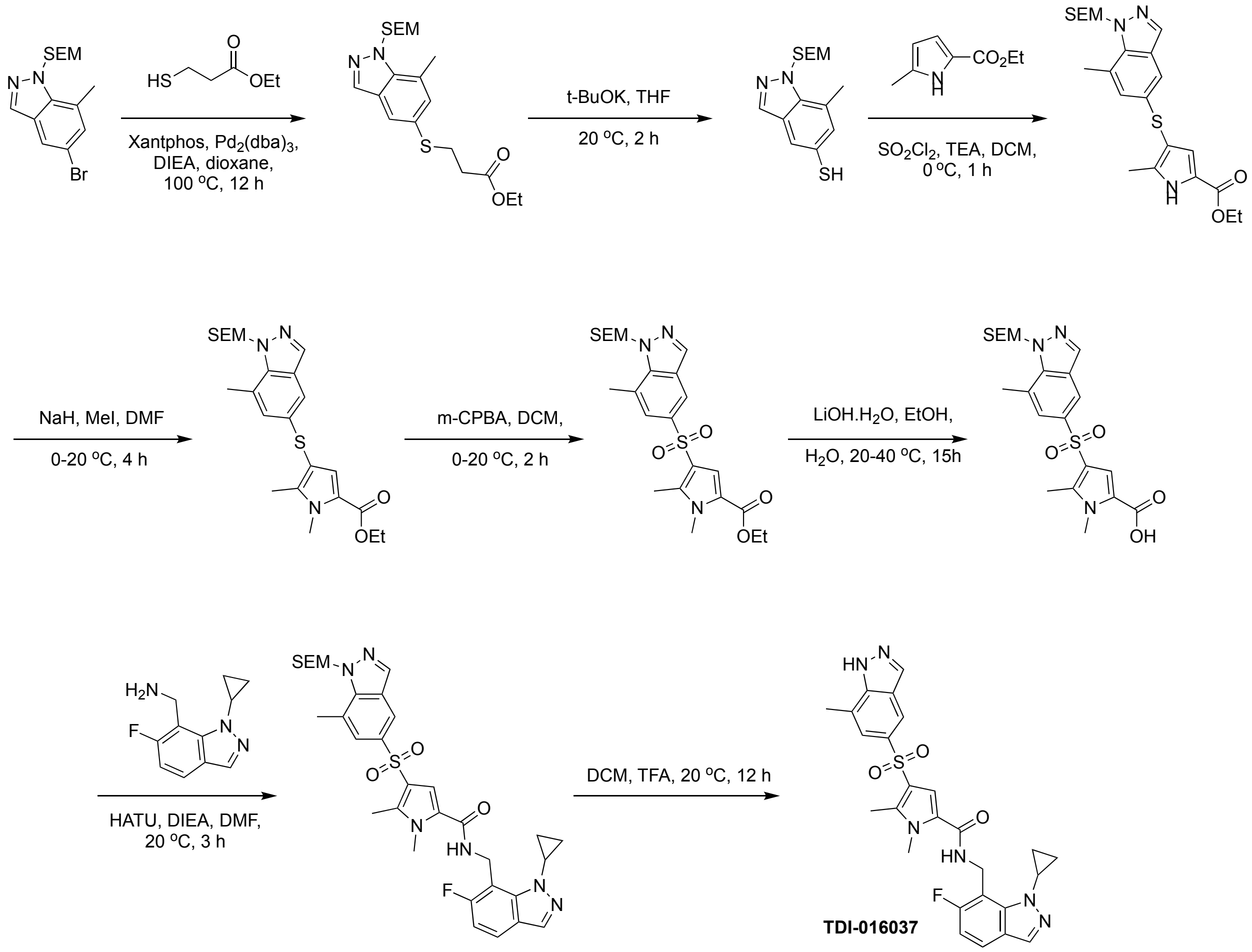

Step 1

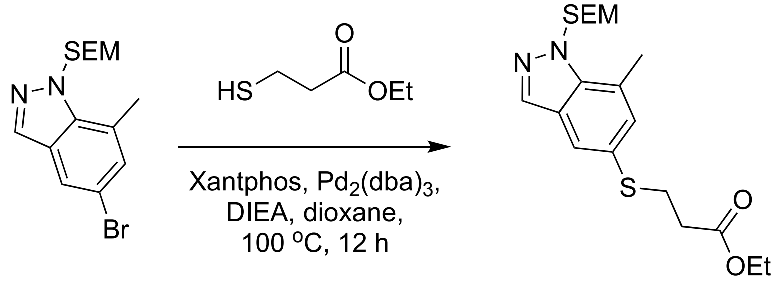

A mixture of 2-[(5-bromo-7-methyl-indazol-1-yl)methoxy]ethyl-trimethyl-silane (7.00 g, 20.5 mmol, 1 *eq*), ethyl 3-sulfanylpropanoate (4.13 g, 30.8 mmol, 1.5 *eq*), DIPEA (7.95 g, 61.5 mmol, 10.7 mL, 3 *eq*), Xantphos (1.19 g, 2.05 mmol, 0.1 *eq*) and $Pd_2(dba)_3$ (1.88 g, 2.05 mmol, 0.1 *eq*) in dioxane (70 mL)was degassed and purged with N_2 for 3 times. The mixture was stirred at 100 °C for 12 h under a N_2 atmosphere. The reaction mixture was diluted with H_2O **(**150 mL), and

the mixture was extracted with EtOAc (80 mL x 3). The combined organic layers were washed with brine (150 mL), dried over Na_2SO_4, and filtered. The filtrate was concentrated under reduced pressure to give a residue. The residue was purified by flash silica gel chromatography (ISCO®; 80 g SepaFlash® silica flash column, eluent of 5 - 30% ethyl acetate/petroleum ether gradient at 60 mL/min) to furnish ethyl 3-[7-methyl-1-(2-trimethylsilylethoxymethyl)indazol-5-yl]sulfanylpropanoate (15.6 g, 22.9 mmol, 56 % yield) as a yellow oil. LCMS (MH^+) 395.2.

Step 2

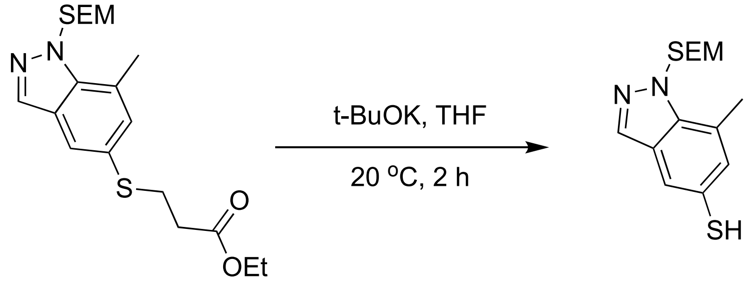

To a solution of ethyl 3-[7-methyl-1-(2-trimethylsilylethoxymethyl)indazol-5-yl] sulfanylpropanoate (15.6 g, 39.5 mmol, 1 *eq*) in THF (160 mL) was added t-BuOK (13.3 g, 119 mmol, 3 *eq*). The mixture was stirred at 20 °C for 2 h. The pH of the reaction mixture was adjusted to 4 by addition of aqueous HCl (2M). The mixture was extracted with EtOAc. The organic layer was concentrated under reduced pressure to give a residue. The residue was purified by flash silica gel chromatography (ISCO®; 220 g SepaFlash® silica flash column, eluent of 11 - 13 % ethyl acetate/petroleum ether gradient at 100 mL/min) to furnish 7-methyl-1-(2-trimethylsilylethoxymethyl) indazole-5-thiol (4.7 g, 15.2 mmol, 38 % yield) as a yellow oil. LCMS (MH^+) 295.1.

Step 3

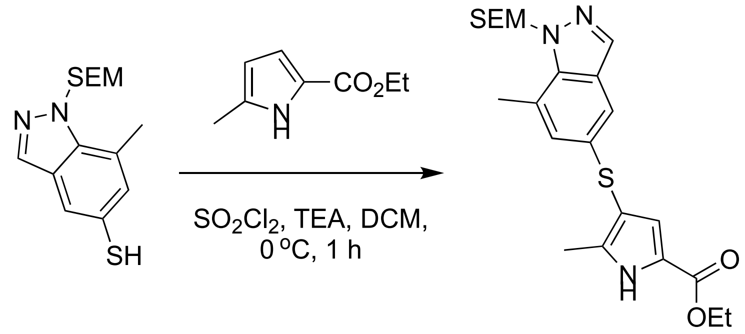

To a solution of 7-methyl-1-(2-trimethylsilylethoxymethyl)indazole-5-thiol (4.70 g, 16.0 mmol, 1 *eq*), TEA (1.62 g, 32.4 mol, 2.23 mL) at 0 °C, a mixture of DCM (60 mL) and sulfuryl chloride (2.59 g, 19.2 mmol, 1.92 mL, 1.2 *eq*) was carefully added in portions. After stirring for 10 minutes, ethyl 5-methyl-1H-pyrrole-2-carboxylate (2.45 g, 16.0 mmol, 1 *eq*) was then added. The resulting solution was stirred for another 50 min. The reaction mixture was concentrated under reduced pressure to remove solvent. The residue was purified by flash silica gel chromatography (ISCO®; 40 g SepaFlash® silica flash column, eluent of 0 - 8% ethyl acetate/petroleum ether gradient at 40 mL/min) to furnish ethyl 5-methyl-4-[7-methyl-1-(2-

trimethylsilylethoxymethyl)indazol-5-yl]sulfanyl-1H- pyrrole-2-carboxylate (3.40 g, 6.64 mmol, 42% yield) was obtained as a yellow oil.

Step 4

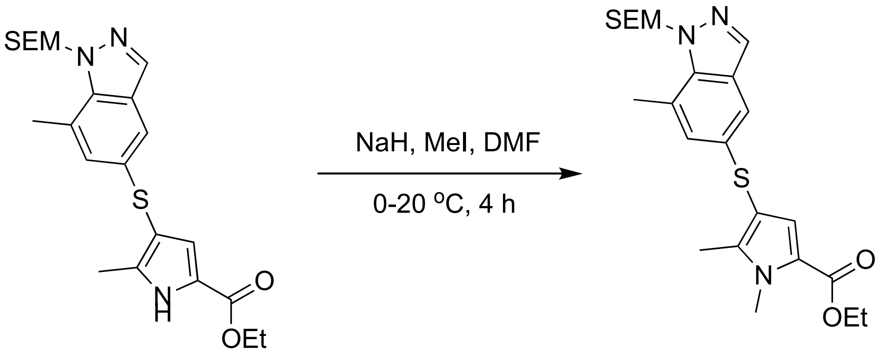

A solution of ethyl 5-methyl-4-[7-methyl-1-(2-trimethylsilylethoxymethyl) indazol-5-yl]sulfanyl-1H-pyrrole-2-carboxylate (3.40 g, 7.63 mmol, 1 *eq*) in DMF (30 mL) at 0 °C was added NaH (610 mg, 15.3 mmol, 60 wt % dispersion in oil) was carefully added in portions. After stirring the mixture for 0.5 h, CH_3I (2.17 g, 15.3 mmol, 2 *eq*) was added. The resulting solution was stirred for another 3.5 h at 20 °C. The reaction mixture was quenched with H_2O (100 mL). The mixture was extracted with EtOAc (50 mL x 3). The combined organic layers were washed with brine (80 mL), dried over Na_2SO_4, and filtered. The filtrate was concentrated under reduced pressure. The residue was purified by flash silica gel chromatography (ISCO®; 40 g SepaFlash® silica flash column, eluent of 0 - 100 % ethyl acetate/petroleum ether gradient at 50 mL/min) to furnish ethyl 1,5-dimethyl-4-[7-methyl-1-(2-trimethylsilylethoxymethyl)indazol-5-yl]sulfanyl-pyrrole-2-carboxylate (3.00 g, 6.53 mmol, 86 % yield) as a yellow solid. LCMS (MH^+) 460.3.

Step 5

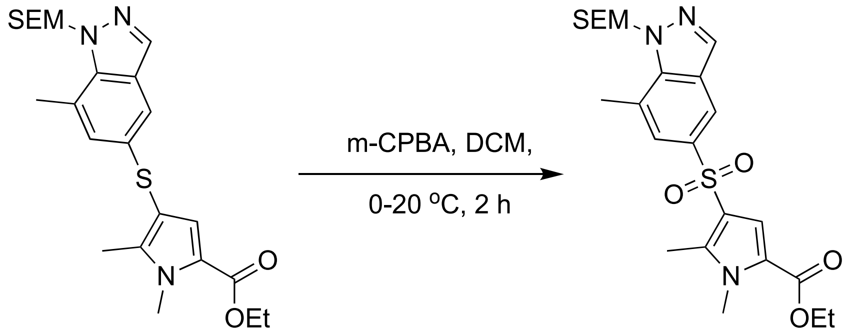

To a solution of ethyl 1,5-dimethyl-4-[7-methyl-1-(2-trimethylsilylethoxymethyl) indazol-5-yl]sulfanyl-pyrrole-2-carboxylate (3.00 g, 6.53 mmol, 1 *eq*) in DCM (30 mL) was added m-CPBA (3.31 g, 16.3 mmol, 85% purity, 2.5 *eq*) at 0°C. The mixture was stirred at 20 °C for 2 h. The reaction mixture was washed sequentially with 10% aqueous Na_2SO_3 (100 mL) and saturated aqueous $NaHCO_3$ (100ml). The organic layer was dried over Na_2SO_4, filtered and concentrated under reduced pressure to give a residue. The residue was purified by flash silica

gel chromatography (ISCO®; 40 g SepaFlash® silica flash column, gradient elution of 0 - 40 % ethyl acetate/petroleum ether at 60 mL/min) to furnish ethyl 1,5-dimethyl-4-[7-methyl-1- (2-trimethylsilylethoxymethyl)indazol-5-yl]sulfonyl-pyrrole-2-carboxylate (2.86 g, 4.13 mmol, 63% yield) as a yellow oil. LCMS (MH^+) 492.2.

Step 6

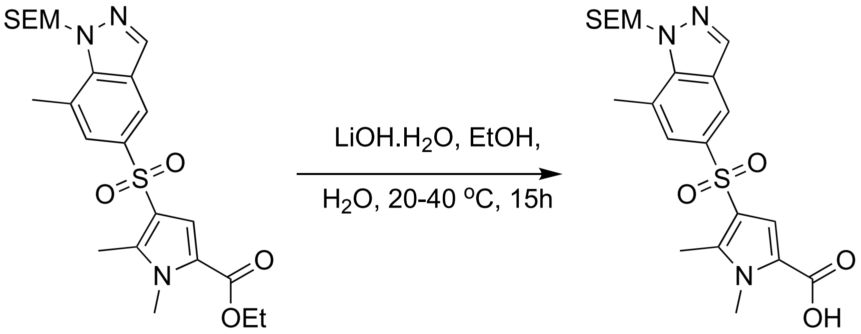

To a solution of ethyl 1,5-dimethyl-4-[7-methyl-1-(2-trimethylsilylethoxymethyl) indazol-5-yl]sulfonyl-pyrrole-2-carboxylate (2.86 g, 5.82 mmol, 1 *eq*) in EtOH (20 mL) and H_2O (4 mL) was added LiOH-H_2O (269 mg, 6.40 mmol, 1.1 *eq*). The mixture was stirred at 20 °C for 12 h. Additional LiOH.H_2O was added (219.69 mg, 5.24 mmol, 0.9 *eq*), and the mixture was stirred at 40 °C for 3 h. The reaction mixture was concentrated under reduced pressure to remove solvent. The residue was diluted with H_2O (25 mL), and the pH was adjusted to 3 by addition of aqueous HCl (2M). The formed solid was filtered and dried under reduced pressure to furnish 1,5-dimethyl-4-[7-methyl-1-(2-trimethylsilylethoxymethyl)indazol-5-yl]sulfonyl- pyrrole-2-carboxylic acid (2.3 g, crude) as a yellow solid. The material was used without further purification. LCMS (MH^+) 464.2.

Step 7

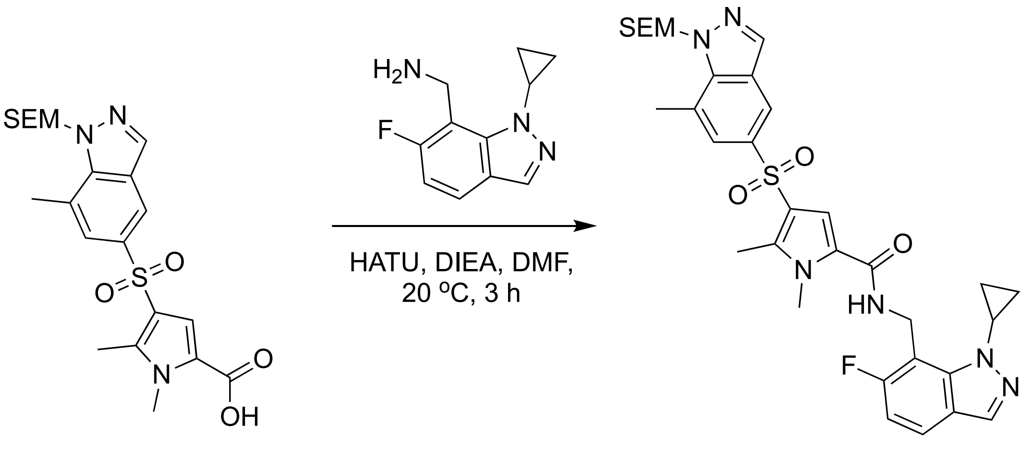

A mixture of 1,5-dimethyl-4-[7-methyl-1-(2-trimethylsilylethoxymethyl)indazol-5-yl] sulfonyl-pyrrole-2-carboxylic acid (100 mg, 0.216 mmol, 1 *eq*), (1-cyclopropyl-6- fluoro-indazol-7-yl)methanamine (44 mg, 0.22 mmol, 1 *eq*), HATU (123 mg, 0.323 mmol, 1.5 *eq*), DIPEA (84 mg, 0.65 mmol, 3 *eq*) in DMF (2.5 mL) was degassed and purged with N_2 (3 x). The mixture

was stirred at 20 °C for 3 h under a N_2 atmosphere. The reaction mixture was diluted with H_2O (20 mL), and the mixture was extracted with EtOAc (10 mL x 3). The combined organic layers were washed with brine (20 mL), dried over Na_2SO_4, and filtered. The filtrate was concentrated under reduced pressure to give a residue. The residue was purified by flash silica gel chromatography (ISCO®; 4 g SepaFlash® silica flash column, gradient elution of 40 - 45 % ethyl acetate/petroleum ether at 40 mL/min) to furnish N-[(1-cyclopropyl-6-fluoro-indazol-7-yl)methyl] -1,5-dimethyl-4-[7-methyl-1-(2-trimethylsilylethoxymethyl)indazol-5-yl]sulfonyl-pyrrole-2-carboxamide (160 mg, 87% purity) as a yellow solid. LCMS (MH^+) 651.3.

Step 8

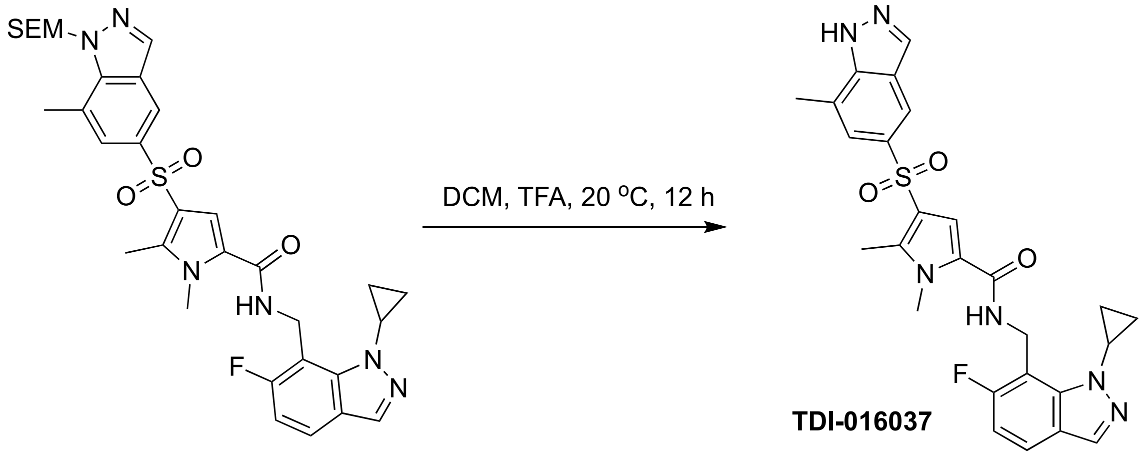

To a solution of N-[(1-cyclopropyl-6-fluoro-indazol-7-yl)methyl]-1,5-dimethyl- 4-[7-methyl-1-(2-trimethylsilylethoxymethyl)indazol-5-yl]sulfonyl-pyrrole-2-carboxamide (140 mg, 0.215 mmol, 1 *eq*) in DCM (3 mL) was added TFA (1 mL) .The mixture was stirred at 20 °C for 12 h. The reaction mixture was concentrated under reduced pressure to remove solvent. The residue was purified by preparative-HPLC (HCl conditions):(column: Phenomenex Luna C18 75 x 30 mm x 3 µm; mobile phase: [water(0.04%HCl)-ACN];gradient: 30%-76% B over 8.0 min) to furnish N- [(1-cyclopropyl-6-fluoro-indazol-7-yl)methyl]-1,5-dimethyl-4-[(7-methyl-1H-indazol-5-yl)sulfonyl]pyrrole-2-carboxamide **TDI-016037** (40 mg)) as a white solid. LCMS (MH^+) 521.0. 1H NMR (400 MHz, DMSO-d6) δ 13.58 (br s, 1H), 8.61 (t, J = 4.3 Hz, 1H), 8.26 (s, 1H), 8.17 (s, 1H), 8.02 (s, 1H), 7.76 (dd, J = 5.1, 8.8 Hz, 1H), 7.43 (s, 1H), 7.22 (s, 1H), 7.07 (dd, J = 8.9, 10.1 Hz, 1H), 4.99 (br d, J = 2.9 Hz, 2H), 3.96 (tt, J = 3.6, 7.1 Hz, 1H), 3.76 (s, 3H), 2.53 (s, 3H), 2.38 (s, 3H), 1.30-1.24 (m, 2H), 1.11-1.02 (m, 2H).

1H NMR of TDI-016037

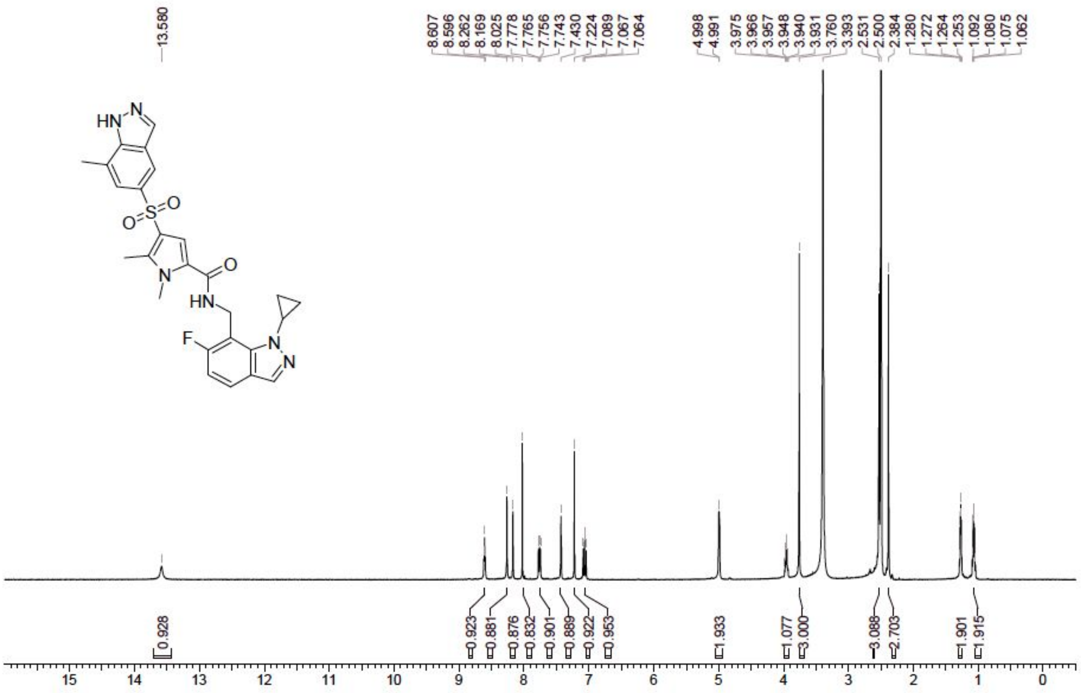

UPLC of TDI-016037

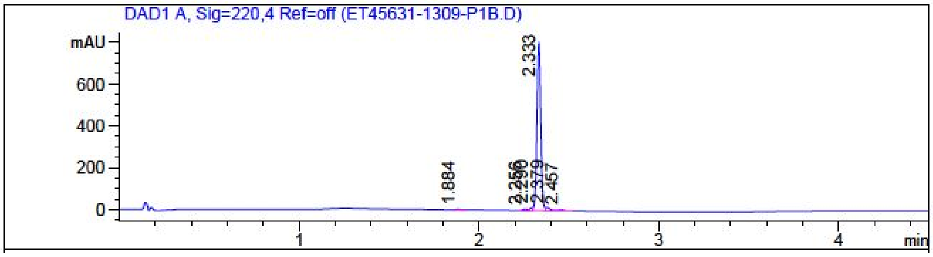

Report

Signal 1 : DAD1 A, Sig=220,4 Ref=off

Peak #	RT [min]	Height	Height %	Width [min]	Area	Area %
1	1.884	3.525	0.412	0.023	5.032	0.381
2	2.256	7.967	0.932	0.024	11.967	0.906
3	2.290	13.451	1.574	0.019	17.560	1.330
4	2.333	808.447	94.583	0.024	1243.749	94.171
5	2.379	16.148	1.889	0.028	30.246	2.290
6	2.457	5.214	0.610	0.033	12.185	0.923

References Supporting Information:

1. Blight, K. J., McKeating, J. A. & Rice, C. M. Highly Permissive Cell Lines for Subgenomic and Genomic Hepatitis C Virus RNA Replication. *J. Virol.* **76,** 13001–13014 (2002).

2. Meyer, C., Garzia, A., Miller, M. W., Huggins, D. J., Myers, R. W., Hoffmann, H.-H., Ashbrook, A. W., Jannath, S. Y., Liverton, N., Kargman, S., Zimmerman, M., Nelson, A. M., Sharma, V., Dolgov, E., Cangialosi, J., Penalva-Lopez, S., Alvarez, N., Chang, C.-W., Oswal, N., Gonzalez, I., Rasheed, R., Goldgirsh, K., Davis, J. A., Ramos-Espiritu, L., Menezes, M.-R., Larson, C., Nitsche, J., Ganichkin, O., Alwaseem, H., Molina, H., Steinbacher, S., Glickman, J. F., Perlin, D. S., Rice, C. M., Meinke, P. T. & Tuschl, T. Small-molecule inhibition of SARS-CoV-2 NSP14 RNA cap methyltransferase. *Nature* **637,** 1178–1185 (2025).

3. Paesen, G. C., Collet, A., Sallamand, C., Debart, F., Vasseur, J.-J., Canard, B., Decroly, E. & Grimes, J. M. X-ray structure and activities of an essential Mononegavirales L-protein domain. *Nat. Commun.* **6,** 8749 (2015).

4. Vuister, G. W., Fogh, R. H., Hendrickx, P. M. S., Doreleijers, J. F. & Gutmanas, A. An overview of tools for the validation of protein NMR structures. *J. Biomol. NMR* **58,** 259–285 (2014).

5. Mysinger, M. M., Carchia, M., Irwin, John. J. & Shoichet, B. K. Directory of Useful Decoys, Enhanced (DUD-E): Better Ligands and Decoys for Better Benchmarking. *J. Med. Chem.* **55,** 6582–6594 (2012).

6. Yang, Y., Yao, K., Repasky, M. P., Leswing, K., Abel, R., Shoichet, B. K. & Jerome, S. V. Efficient Exploration of Chemical Space with Docking and Deep Learning. *J. Chem. Theory Comput.* **17,** 7106–7119 (2021).

7. Heinzelmann, G., Huggins, D. J. & Gilson, M. K. BAT2: an Open-Source Tool for Flexible, Automated, and Low Cost Absolute Binding Free Energy Calculations. *J. Chem. Theory Comput.* **20,** 6518–6530 (2024).

8. Huggins, D. J. Comparing the Performance of Different AMBER Protein Forcefields, Partial Charge Assignments, and Water Models for Absolute Binding Free Energy Calculations. *J. Chem. Theory Comput.* **18,** 2616–2630 (2022).

9. Aldeghi, M., Heifetz, A., Bodkin, M. J., Knapp, S. & Biggin, P. C. Accurate calculation of the absolute free energy of binding for drug molecules. *Chem. Sci.* **7,** 207–218 (2015).

10. Qian, Y., Vaca, I. C. de, Vilseck, J. Z., Cole, D. J., Tirado-Rives, J. & Jorgensen, W. L. Absolute Free Energy of Binding Calculations for Macrophage Migration Inhibitory Factor in Complex with a Druglike Inhibitor. *J. Phys. Chem. B* **123,** 8675–8685 (2019).

11. Fu, H., Gumbart, J. C., Chen, H., Shao, X., Cai, W. & Chipot, C. BFEE: A User-Friendly Graphical Interface Facilitating Absolute Binding Free-Energy Calculations. *J. Chem. Inf. Model.* **58,** 556–560 (2018).

12. Rocklin, G. J., Mobley, D. L. & Dill, K. A. Separated topologies—A method for relative binding free energy calculations using orientational restraints. *J. Chem. Phys.* **138,** 085104 (2013).

13. Baumann, H. M., Dybeck, E., McClendon, C. L., Pickard, F. C., Gapsys, V., Pérez-Benito, L., Hahn, D. F., Tresadern, G., Mathiowetz, A. M. & Mobley, D. L. Broadening the Scope of Binding Free Energy Calculations Using a Separated Topologies Approach. *J. Chem. Theory Comput.* **19,** 5058–5076 (2023).

14. Debiec, K. T., Cerutti, D. S., Baker, L. R., Gronenborn, A. M., Case, D. A. & Chong, L. T. Further along the Road Less Traveled: AMBER ff15ipq, an Original Protein Force Field Built on a Self-Consistent Physical Model. *J. Chem. Theory Comput.* **12,** 3926–3947 (2016).

15. Jorgensen, W. L., Chandrasekhar, J., Madura, J. D., Impey, R. W. & Klein, M. L. Comparison of simple potential functions for simulating liquid water. *J. Chem. Phys.* **79,** 926–935 (1983).

16. Wang, J., Wolf, R. M., Caldwell, J. W., Kollman, P. A. & Case, D. A. Development and testing of a general amber force field. *J. Comput. Chem.* **25,** 1157–1174 (2004).

17. Jakalian, A., Jack, D. B. & Bayly, C. I. Fast, efficient generation of high-quality atomic charges. AM1-BCC model: II. Parameterization and validation. *J. Comput. Chem.* **23,** 1623–1641 (2002).

18. Sun, S. & Huggins, D. J. Assessing the effect of forcefield parameter sets on the accuracy of relative binding free energy calculations. *Front. Mol. Biosci.* **9,** 972162 (2022).

19. Boresch, S., Tettinger, F., Leitgeb, M. & Karplus, M. Absolute Binding Free Energies: A Quantitative Approach for Their Calculation. *J. Phys. Chem. B* **107,** 9535–9551 (2003).

20. Eastman, P., Swails, J., Chodera, J. D., McGibbon, R. T., Zhao, Y., Beauchamp, K. A., Wang, L.-P., Simmonett, A. C., Harrigan, M. P., Stern, C. D., Wiewiora, R. P., Brooks, B. R. & Pande, V. S. OpenMM 7: Rapid development of high performance algorithms for molecular dynamics. *PLoS Comput. Biol.* **13,** e1005659 (2017).

21. Hopkins, C. W., Grand, S. L., Walker, R. C. & Roitberg, A. E. Long-Time-Step Molecular Dynamics through Hydrogen Mass Repartitioning. *J. Chem. Theory Comput.* **11,** 1864–1874 (2015).

22. Darden, T., York, D. & Pedersen, L. Particle mesh Ewald: An N ·log(N) method for Ewald sums in large systems. *J. Chem. Phys.* **98,** 10089–10092 (1993).

23. Pham, T. T. & Shirts, M. R. Identifying low variance pathways for free energy calculations of molecular transformations in solution phase. *J. Chem. Phys.* **135,** 034114 (2011).

24. Shirts, M. R. & Chodera, J. D. Statistically optimal analysis of samples from multiple equilibrium states. *J. Chem. Phys.* **129,** 124105 (2008).
